# Survival-Associated Cellular Response Maintained in Pancreatic Ductal Adenocarcinoma (PDAC) Switched Between Soft and Stiff 3D Microgel Culture

**DOI:** 10.1021/acsbiomaterials.3c01079

**Published:** 2024-03-11

**Authors:** Dixon J. Atkins, Jonah M. Rosas, Lisa K. Månsson, Nima Shahverdi, Siddharth S. Dey, Angela A. Pitenis

**Affiliations:** † Department of Biomolecular Science and Engineering, University of California Santa Barbara, Santa Barbara, California 93106, United States; ‡ Materials Department, University of California Santa Barbara, Santa Barbara, California 93106, United States; § Molecular, Cellular, and Developmental Biology Department, University of California Santa Barbara, Santa Barbara, California 93106, United States; ∥ Department of Chemical Engineering, University of California Santa Barbara, Santa Barbara, California 93106, United States; ⊥ Department of Bioengineering, University of California Santa Barbara, Santa Barbara, California 93106, United States

**Keywords:** mechanical memory, 3D cell culture, confinement, stiffness, microgel

## Abstract

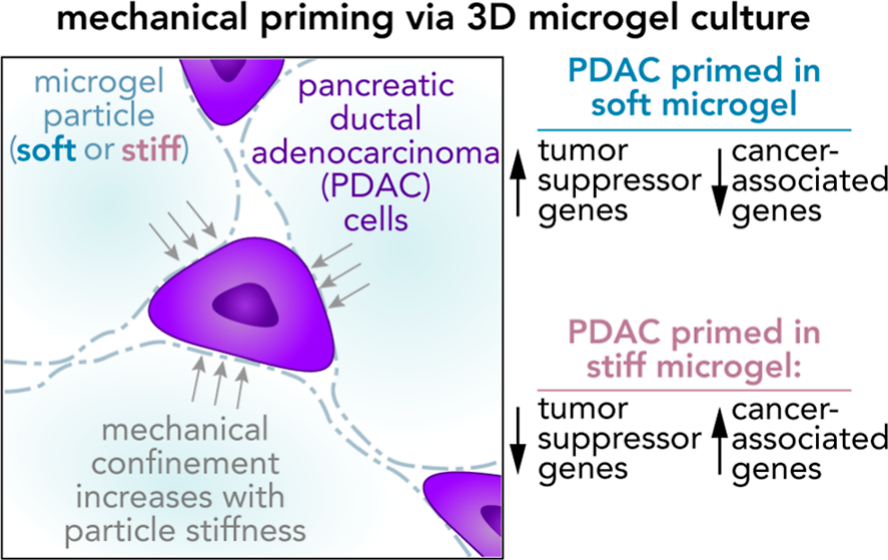

Pancreatic ductal adenocarcinoma (PDAC) accounts for about 90% of all pancreatic cancer cases. Five-year survival rates have remained below 12% since the 1970s, in part due to the difficulty in detection prior to metastasis (migration and invasion into neighboring organs and glands). Mechanical memory is a concept that has emerged over the past decade that may provide a path toward understanding how invading PDAC cells “remember” the mechanical properties of their diseased (“stiff”, elastic modulus, *E* ≈ 10 kPa) microenvironment even while invading a healthy (“soft”, *E* ≈ 1 kPa) microenvironment. Here, we investigated the role of mechanical priming by culturing a dilute suspension of PDAC (FG) cells within a 3D, rheologically tunable microgel platform from hydrogels with tunable mechanical properties. We conducted a suite of acute (short-term) priming studies where we cultured PDAC cells in either a soft (*E* ≈ 1 kPa) or stiff (*E* ≈ 10 kPa) environment for 6 h, then removed and placed them into a new soft or stiff 3D environment for another 18 h. Following these steps, we conducted RNA-seq analyses to quantify gene expression. Initial priming in the 3D culture showed persistent gene expression for the duration of the study, regardless of the subsequent environments (stiff or soft). Stiff 3D culture was associated with the downregulation of tumor suppressors (*LATS1*, *BCAR3*, *CDKN2C*), as well as the upregulation of cancer-associated genes (*RAC3*). Immunofluorescence staining (BCAR3, RAC3) further supported the persistence of this cellular response, with BCAR3 upregulated in soft culture and RAC3 upregulated in stiff-primed culture. Stiff-primed genes were stratified against patient data found in The Cancer Genome Atlas (TCGA). Upregulated genes in stiff-primed 3D culture were associated with decreased survival in patient data, suggesting a link between patient survival and mechanical priming.

## 1 Introduction

**Figure 1:**
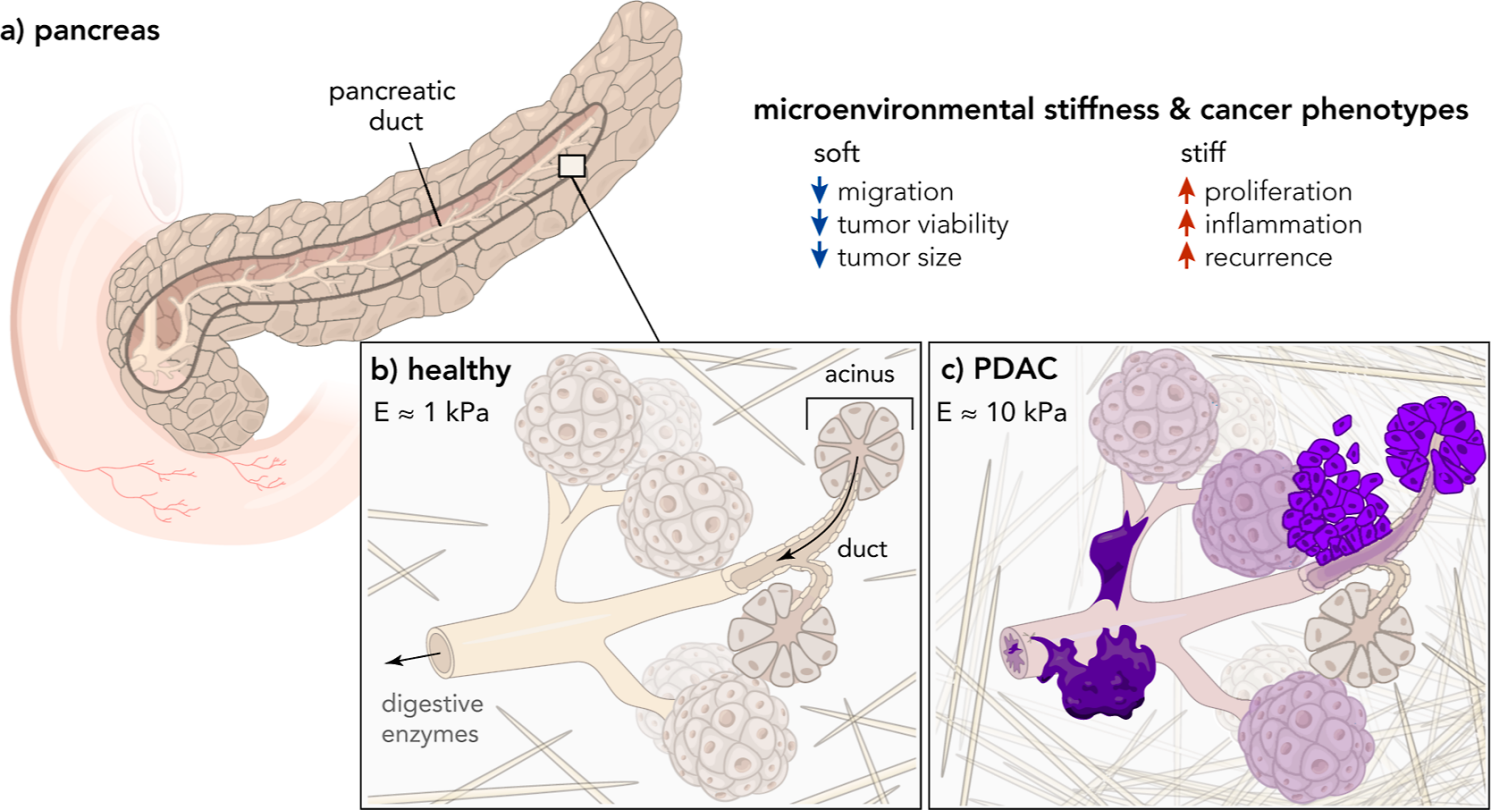
(a) The pancreas is a gland about 15 cm in length located in the abdomen and is involved with the secretion of digestive enzymes. (b) About 90% of cancers of the pancreas arise from the acinar cells that secrete these digestive enzymes and/or the ductal cells used in the transport of these enzymes. In healthy pancreatic tissue, ECM proteins are dispersed and unaligned, resulting in a soft microenvironment.^4^ (c) Pancreatic ductal adenocarcinomas develop desmoplasia, or the growth of fibrous tissue, around the tumor site. These fibrous tissue growths are associated with an increase in the stiffness surrounding and within the tumor microenvironment due to increased alignment and density of the ECM.^4^ Softer microenvironments have been associated with decreases in migration, better chemotherapeutic outcomes, and smaller tumor sizes, while stiffer microenvironments have been correlated with increases in inflammatory markers, cell proliferation, and recurrence.^6−11^

Pancreatic cancer is a devastating and insidious disease. Many cases arise when cells within the pancreas divide uncontrollably, form masses, and invade nearby organs and glands before diagnosis. The American Cancer Society estimates that in 2023, about 64,050 new cases will be diagnosed in the United States and 50,550 will succumb to the disease.^1^ Across all stages, the 5 year survival rate is 12%, among the lowest survival rates for all cancers in the United States.^1^ Early stages are not typically accompanied by symptoms, and diagnosis typically occurs after cancer has spread to distant locations. While pancreatic cancer manifests in many forms, pancreatic ductal adenocarcinoma (PDAC) accounts for about 90% of cases.^2^ This type of pancreatic cancer originates in the cells responsible for producing and transporting digestive enzymes (Figure 1). The transition from healthy to cancerous pancreatic tissue correlates with increased extracellular matrix (ECM) deposition and desmoplasia, which also corresponds to increased elastic modulus from about *E* = 1 kPa (healthy tissue) to *E* = 10 kPa (PDAC tissue).^3,4^ Clinical observations suggest that once a tumor approaches the resection margin, even if it has not metastasized, the prognosis is poor.^5^

We hypothesize that following migration and invasion, PDAC cells “remember” the mechanical microenvironment of their original tumor via “mechanical memory”. The concept of mechanical memory was first put forth by Balestrini et al., who defined it as cells’ ability to “permanently imprint information regarding substrate mechanical conditions”.^16^ Mechanical memory has been implicated in migration, proliferation, and many other cancer-associated phenotypes (Table 1). Table 1 contains a compilation of mechanical priming studies over the past decade from a Web of Science query focused on “mechanical memory”, although this area of research is rapidly expanding, and this list is not exhaustive. To the authors’ knowledge, few in vitro investigations into the mechanical memory of pancreatic cancer have been carried out in either 2D or 3D.^7,15^

**Table 1:** Key Studies Focused on Mechanical Memory^a^

priming dimension (2D/3D)	priming stiffness (kPa)	final stiffness (kPa)	priming duration (days)	cell type	behavior related to mechanical memory	ref
2D	0.5	50	1–3	MCF10A healthy breast epithelia	stiff priming led to faster migration and upregulation of contractile proteins when switched to a softer matrix	Nasrollahi et al., 2017
	50	0.5				
	0.5	20	5	Cal27 squamous cell carcinoma	stiff substrates resulted in faster migration and higher EMT markers and myosin. Transcriptomic changes on stiff substrates corresponded to poor patient prognosis	Moon et al., 2023
	20	0.5				
	0.5	8.0	7	SUM159 mesenchymal triple-negative breast cancer	stiff-conditioned cells formed larger tumors in mouse bone marrow	Watson et al., 2021
	8.0	0.5				
	10	100	12	SUIT-2.28 pancreatic cancer	soft-primed cells showed lower YAP nuclear translocation and a loss of rigidity sensing through YAP	Carnevale et al., 2019
	100	10				
	5	25–100	14–21	primary rat fibroblasts	stiff-primed cells had higher fibrotic activity on soft substrates. Soft primed cells had diminished fibrotic activity	Balestrini et al., 2012
	100	5				
	100	1	20	hASC adipose-derived stem cell	stiff substrates reduced adipogenesis	Berger et al., 2021
	5	100				
	1	120	14–28	ASC primary adipose-derived stem cell	soft priming decreased cell area, actin coherency, and ECM production	Dunham et al., 2020
	120	1				
	5	100	21–35	MSC mesenchymal stem cells	soft substrates suppress fibrogenesis and desensitize MSCs	Li et al., 2017
	100	5				
	2	10	1–10	hMSC human mesenchymal stem cells	YAP/TAZ and RUNX2 were irreversibly activated by stiff substrates; stiff substrates directed stem cell fate toward osteogenic differentiation	Yang et al., 2014
	10	2				
	10	100	7	hMSC human mesenchymal stem cells	stiff conditions promoted osteogenic differentiation, which was diminished but not completely removed when transitioned to soft conditions	Wei et al., 2020
3D	100	10				
	0.75	0.15	7	COLO-357 human pancreatic cancer cells	stiffening matrices conferred control of cell spheroid growth and promoted drug resistance	Arkenberg et al., 2018
	0.15	0.75				

a Most studies were conducted by priming cells in 2D culture, over a wide range of stiffnesses, and priming duration was typically on the order of days.^7,12−21^

In this study, we developed a 3D in vitro platform to investigate the survival-associated cellular response in PDAC switched between soft and stiff microenvironments. The effects of both 3D stiffness and confinement play an integral role in directing cellular response and mechanical memory. Priming duration typical of 2D mechanical memory studies may not translate to 3D systems, where cells experience decreased contractility and increased cellular confinement.^22^ While most investigations have focused on long-term mechanical memory, we examined acute conditions (<24 h) to simulate recently migrated cancer cells. We explored the effects of confinement and mechanical priming of dilute suspensions of PDAC cells within a 3D polyacrylamide microgel support medium. These microgels were formed from polyacrylamide hydrogels which have been shown to support 3D cell culture without the need for adhesive proteins.^23−25^ We used RNA-seq to evaluate the gene expression profiles of cell populations that were primed in either a soft or stiff 3D microgel culture. PDAC cells were primed for 6 h in either soft or stiff environments to capture the short time frame during which previous investigations have shown widespread mRNA changes^26^ and basement membrane perforation.^27^ In confinement, nuclear deformation likely induces mechanosensation and changes in gene expression.^28−31^ Our platform opens up new opportunities to investigate 3D in vitro models of pancreatic cancer and probing the fundamental mechanisms of mechanical memory.

## 2 Experimental

### 2.1 Microgel Platform

Polyacrylamide hydrogels were prepared using two different compositions: (1) 3 wt % acrylamide, 0.12 wt % *N*,*N*′-methylene bis(acrylamide) (MBAm), 0.15 wt % tetramethylethylenediamine (TEMED), and 0.15 wt % ammonium persulfate (APS) in ultrapure water (18.2 MΩ resistivity); and (2) 5 wt % acrylamide, 0.2 wt % *N*,*N*′-methylenebis(acrylamide) (MBAm), 0.15 wt % tetramethylethylenediamine (TEMED), and 0.15 wt % ammonium persulfate (APS) in ultrapure water (18.2 MΩ resistivity). Bulk hydrogels polymerized in two 50 mL polystyrene conical vials (100 mL total) and equilibrated in ultrapure water for at least 24 h before they were mechanically fragmented into 110 μm diameter microgel particles (Figure S1) following previous methods.^25,32^ Our methods are also similar to previously established extrusion fragmentation protocols.^25,32−36^ Microgel particles were classified by size using Stokes’ sedimentation and quantified with microscopy (Figure S2). Solutions of about 50 vol % microgel particles were equilibrated in cell culture media for at least 24 h prior to testing.

### 2.2 Mechanical Properties of Bulk Hydrogels

Previous nanomechanical measurements of microscale polyacrylamide hydrogel spheres^37^ resulted in similar values of elastic modulus (between 1 and 10 kPa) as those determined from micromechanical measurements of millimeter-scale polyacrylamide hydrogels with similar water content and cross-link density.^38^ Here, a custom-built microtribometer^39−43^ was used for microindentation measurements of bulk hydrogels prior to fragmentation. Hydrogel samples were equilibrated in ultrapure water for at least 24 h prior to micromechanical testing. Hemispherical glass probes (radius of curvature *R* = 2.6 mm) affixed to a double-leaf cantilever with a spring constant in the normal direction of *K*_n_ = 210 μN/μm indented bulk hydrogel samples to a maximum normal force of 750 μN at a constant indentation velocity *v* = 1 μm/s. During indentation measurements, probes and bulk hydrogel samples were fully submerged in ultrapure water.

Reduced elastic modulus, E*, for each hydrogel was calculated by fitting experimental data to the Hertzian contact mechanics model from the normal force, *F*_n_, radius of curvature, *R*, and microindentation depth, *d* (eq 1).
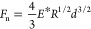
1

### 2.3 Microgel Rheology

The TA Instruments ARES-G2 rotational rheometer measured the viscoelasticity of the microgel 3D culture system. Microgel particles were centrifuged with a Thermo Scientific ST8R Refrigerated Benchtop Centrifuge at 1200*g* with soft deceleration for 10 min to increase packing fraction to about 60% prior to rheological testing.^44^ Microgel was dispensed between two parallel aluminum plates (25 mm diameter and 500 μm gap height). Storage moduli of both soft and stiff microgels were individually evaluated from 0.63 to 63 rad/s angular frequencies at 1% strain amplitude. For both soft and stiff microgels, storage and loss moduli were measured from 0.1 to 1000% oscillatory strain amplitude at a frequency of 1 Hz.

### 2.4 Microgel Packing Density Analysis

Microgel were prepared according to the aforementioned protocol. 100 nm Fluoro-Max Dyed Green Aqueous Fluorescent Particles (Thermo Scientific Cat. no. G100) were diluted to a concentration of 1%, mixed with microgel particles, and centrifuged at 1200*g* for 10 min. Confocal images were taken using a Nikon A1R HD confocal microscope with a 10× objective (NA = 0.30) and 2.25 μm z-stacks. Local thickness was calculated using an overlapping ball algorithm (Local Thickness) built into FIJI, and mean and standard deviations were calculated across each z-slice.

### 2.5 2D Cell Culture

Human pancreatic ductal adenocarcinoma cells (FG, a well-differentiated PDAC line that harbors mutated KRAS^G12D^, kindly gifted by the Reya Lab)^45,46^ were cultured on standard polystyrene flasks (Fisherbrand Surface Treated Sterile Tissue Culture Flasks, Vented Cap, Cat. no. FB012937) in normal growth media consisting of DMEM, high glucose, GlutaMAX Supplement (Gibco, Cat. no. 10569044), and 10% fetal bovine serum (Gibco, Cat. no. 16000044), 1% Penicillin–Streptomycin (Gibco, Cat. no. 15140122), and 1% nonessential amino acids (Gibco, Cat. no. 11140050). Cells were thawed from cryogenic storage and used within 5 passages. Cells were maintained in an incubator at 37 °C, 5% CO_2_, and 95% relative humidity and passaged before reaching 80% confluence (every 2 to 3 days).

### 2.6 Immunofluorescence Assays

After 24 h of culture in soft or stiff microgel, media was aspirated from the microgel particles and cells, and the microgel and cells were transferred to 1.5 mL Eppendorf tubes. Cells were fixed using 1 mL of 4% PFA in PBS per 1.5 mL Eppendorf tube for 15 min at 25 °C and washed three times with 1× PBS for 10 min. Cells were permeabilized using 0.5% Tween-20 for 30 min at 25 °C and then washed three times with 1× PBS for 10 min. Cells were blocked with 10% normal goat serum (NGS) for 45 min at 25 °C before primary antibody incubation at 1:250 dilution for 12 h at 4 °C. Primary antibodies used were acetyl-alpha Tubulin (Lys40) (Thermo Fisher, cat. no. 32-2700), BCAR3 (ThermoFisher, cat. no. PA5-101074), and RAC3 (Thermo Fisher, Cat. no. 16117-1-AP). Samples were washed three times with 1× PBS for 10 min, and secondary antibodies (Alexa Fluor goat antimouse 488/Alexa Fluor goat antirabbit 488, 1:250, along with Hoechst 1:500 and Phalloidin 1:500) were incubated with cells for 1 h at 25 °C. Samples were mounted between coverslips and glass slides in Fluoromount, sealed, and allowed to cure for 24 h before imaging. Representative images were taken using a Nikon A1R HD Confocal and 60× (NA = 1.40) objective. Fluorescence intensity values were calculated from sum-*z*-intensity projections taken using a 20× (NA = 0.45) objective. Total fluorescence values were normalized to cell area according to eq 2.
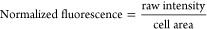
2

Tubulin expression data were quantified as normalized fluorescence across a radial cross section. A 6.5 μm average-intensity z-projection was formed over the midplane of the cell, identified by the largest presence of the nucleus. Intensity values were measured across the *x*-plane and normalized to the cell diameter length and maximum fluorescence intensity to show differences in localization.

### 2.7 3D Cell Culture

Microgels used in the 3D culture were equilibrated for at least 24 h in cell culture media. Three wells of a 6-well plate were filled with soft microgel, and the other three wells were filled with stiff microgel. Each well contained a volume of microgel of about 667 μL which was roughly 2 mm high and about 20 mm in diameter. The filled 6-well plate was centrifuged with a Thermo Scientific ST8R Refrigerated Benchtop Centrifuge at 1200*g* with soft deceleration for 10 min to increase the packing fraction of microgel (about 60 vol %).

Cells were detached from standard polystyrene flasks using trypsin and pelleted. Cells were resuspended in microgel to a 10 vol % mixture of cells in microgel. This mixture was used to print 8 individual 1 μL cell suspensions (about 25,000 cells) in multiple locations per well. Low concentrations of cells were supported by and confined within the 3D microgel culture (see confocal images, Figure S3). Each dilute cluster of cells in microgel was deposited in the middle of the 2 mm high microgel layer, and away from rigid boundaries, to ensure cells remained within a 3D microenvironment. Each well was filled with 2 mL of fresh culture media and placed in an incubator for 6 h at 37 °C, 5% CO_2_, and 95% relative humidity. Cells, microgel, and media were transferred to 15 mL conical vials and centrifuged at 300*g* for 10 min. After 6 h, at least 100,000 cells were extracted from each of the soft and stiff microgel conditions for RNA sequencing. The remaining cells in each well were extracted for continued mechanical priming experiments (soft-primed, stiff-primed).

Eight wells across two 6-well plates were prepared with microgel (four soft, four stiff) as previously described. At least 100,000 of the previously extracted cells (post 6 h in 3D culture; either soft-primed or stiff-primed) were deposited into each of these new microgel-filled wells. Wells that contained stiff microgels received cells that had been cultured for 6 h in either soft microgel (soft-to-stiff) or stiff microgel (stiff-to-stiff). The same procedure was used for new wells containing soft microgel (soft-to-soft and stiff-to-soft). Each well was filled with 2 mL of fresh culture media and placed in an incubator at 37 °C, 5% CO_2_, and 95% relative humidity for 18 h, after which at least 100,000 cells were collected for RNA sequencing. Cell viability was above 80% for both soft and stiff microgel cultures after 24 h (Figure S4).

### 2.8 RNA Sequencing and Statistical Analyses

Total RNA was extracted using TRIzol reagent (Invitrogen) in accordance with the manufacturer’s protocol (Pub. no. MAN0001271C.0). Bulk mRNA sequencing using the CEL-Seq2 technique was performed on 100 ng of total RNA per sample according to previously published protocol.^47^ Briefly, mRNA fragments were randomly primed and reverse transcribed into cDNA. The second cDNA strand was synthesized using dNTPs, *E. coli* DNA Polymerase I (Invitrogen), first strand buffer (Invitrogen), and RNase H (Thermo Scientific). DNA fragments were purified using DNA magnetic beads (AMPure). cDNA then underwent in vitro transcription to linearly amplify the product. This amplified RNA was then fragmented and reverse transcribed into cDNA again. The second-strand cDNA was ligated to Illumina sequencing adapters and amplified via PCR amplification and the size was selected with two 0.8× DNA bead cleanups (AMPure).

DNA libraries were sequenced using the NovaSeq 6000 system (Illumina), and raw reads were normalized and mapped to the human reference genome release hg19 (GRCh37).^48^ RNA-seq raw counts were modeled parametrically assuming a negative binomial distribution with the *DESeq2* package to determine differentially expressed genes.^49^ The p-values of differentially expressed genes were adjusted using the Benjamini and Hochberg method.^49^ The R package *ashr* was used to calculate the log fold change shrinkage.^50^ Two biological repeats were analyzed for differential expression analysis under the 6 h primed conditions, and two technical replicates of two biological repeats were used in the differential expression analysis conducted after 24 h. The R package *LIMMA* was used to remove batch effects.^51^ Hierarchical clustering was carried out using the *ComplexHeatmap* function in R to identify groups in the data set.^52^ We calculated Pearson correlation coefficients using the R package *psych* to show correlations among repeats (Figure S5).^53^ We performed systems level data set analysis to identify gene ontology terms.^54^

## 3 Results and Discussion

### 3.1 Mechanical Characterization of Hydrogels

**Figure 2:**
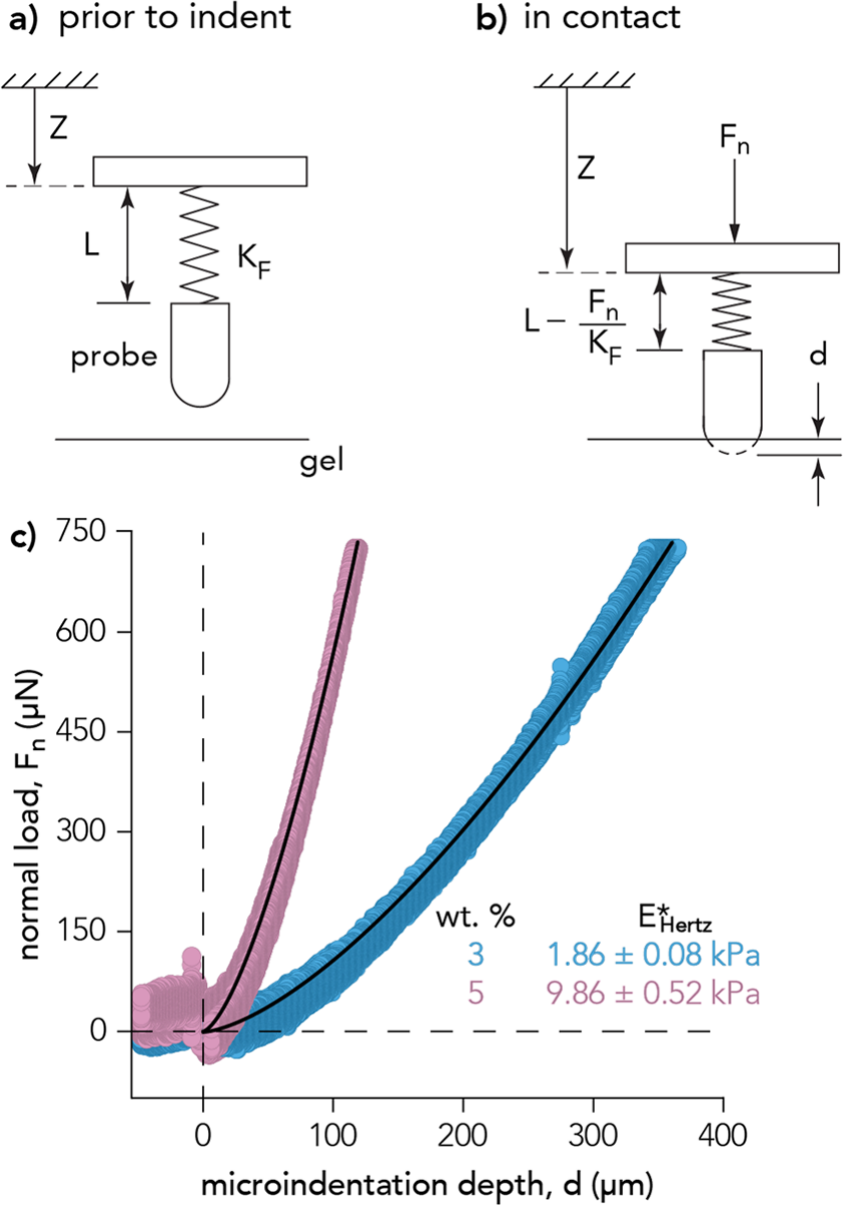
(a) Schematic of microindentation instrument prior to contact with bulk hydrogel. Displacement of the vertical piezoelectric nanopositioning stage, *Z*, the initial distance of the cantilever flexure from the vertical stage, *L*, and the spring constant of the flexure, *K*_F_ are shown. (b) During contact, the vertical stage displacement and flexure compression result in normal force (*F*_n_) applied until the glass probe has reached some microindentation depth within the bulk hydrogel, *d*. (c) Force-displacement curves (approach and retraction) of soft 3 wt % (blue circles) and stiff 5 wt % (pink circles) polyacrylamide bulk hydrogels indented at *v* = 1 μm/s to a maximum normal force of *F*_n_ = 750 μN. The average and standard deviation of the reduced elastic modulus, E*, are calculated from the Hertzian contact mechanics fit (solid black lines) of the approach curve using 3 individual bulk hydrogels and 4 locations per sample, over a total of 12 indents (*n* = 12).

Polyacrylamide hydrogels were tuned to mimic the mechanical microenvironment of the healthy pancreas and that of pancreatic ductal adenocarcinoma.^4^ The material properties of bulk polyacrylamide hydrogels were evaluated using microindentation (Figure 2a,b). The Hertzian contact mechanics model (eq 1) was used to fit the approach curves (Figure 2c). Microindentation measurements resulted in reduced elastic moduli, E* = 1.86 ± 0.08 kPa (soft, 3 wt % bulk polyacrylamide hydrogel) and E* = 9.86 ± 0.52 kPa (stiff, 5 wt % bulk polyacrylamide hydrogel). These results align well with the reported elastic moduli of healthy and cancerous pancreatic tissues.^4^

### 3.2 Rheological Characterization of Microgel

**Figure 3:**
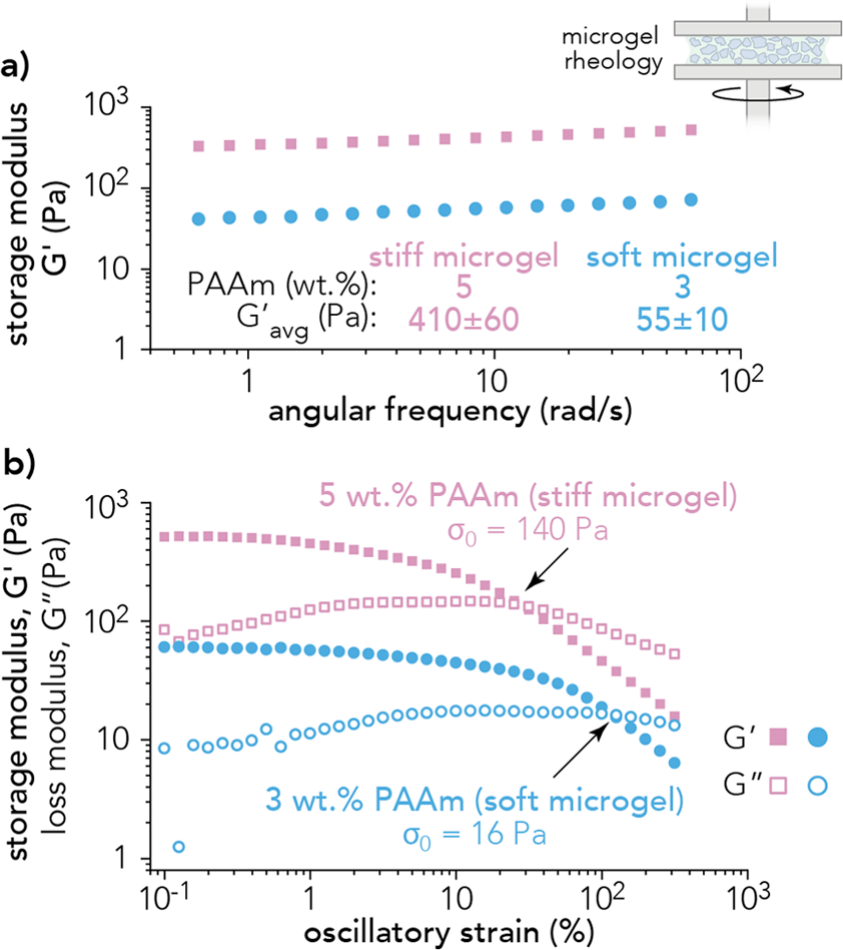
(a) Microgel rheology was performed between two aluminum parallel plates (25 mm diameter) with a 500 μm gap height. Frequency sweeps show the linear plateau of the storage modulus, *G*′. For the soft microgel (blue), the average storage modulus was *G*′ = 55 ± 10 Pa. For the stiff microgel (pink), the average storage modulus was *G*′ = 410 ± 60 Pa. (b) A strain sweep was performed at 1 Hz on jammed microgel (110 μm diameter particles) of either 3 wt % (blue) or 5 wt % polyacrylamide (pink). Both microgel formulations exhibit yielding at the storage modulus (*G*′) and loss modulus (*G*″) crossover point. (*n* = 2).

The rheological properties of soft and stiff microgels were analyzed using a parallel plate rheometer. The packing fraction and average channel width of the soft and stiff microgel systems were negligible. These microgel systems had packing fractions of 61 ± 3% for the soft microgel and 57 ± 3% for the stiff microgel (Figure S6). Furthermore, channel widths between microgel particles were calculated as 11.7 ± 21 and 8.5 ± 14 μm for the soft and stiff microgel, respectively (Figure S7). These length scales are of the same order of magnitude as the diameter of the PDAC cells in this study. The storage modulus, *G*′, of both soft and stiff PAAm microgels present linear viscoelastic regions up to an angular frequency of 10 rad/s (Figure 3a). Since the linear viscoelastic region spans 0.5 to 500 rad/s, the jammed microgel exhibits solid-like properties at low strains and low frequencies. The average storage moduli in the linear viscoelastic region were *G*_soft_′ = 55 ± 10 Pa (soft microgel) and *G*_stiff_′ = 410 ± 60 Pa (stiff microgel). Strain sweeps were conducted to determine the yield stresses of the soft and stiff microgels. Briefly, when the storage modulus, *G*′, crosses over the loss modulus, *G*″, the material has yielded, and the microgel exhibits fluid-like properties. Stiff microgel exhibits a yield stress of σ_0,stiff_ = 140 Pa, and soft microgel exhibits a yield stress of σ_0,soft_ = 16 Pa (Figure 3b).

### 3.3 3D Microgel Mechanics Direct Changes in Gene Expression

**Figure 4:**
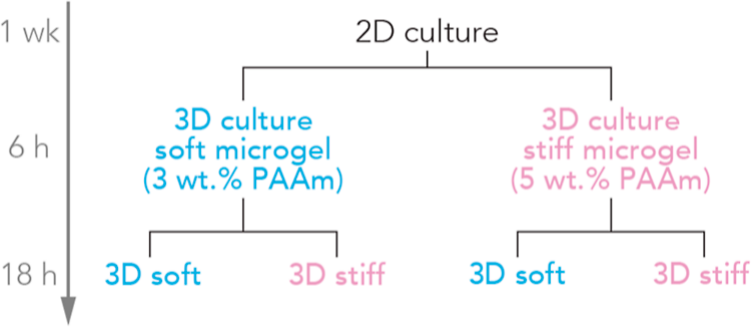
Experimental procedure for probing transcriptomic maintenance related to the mechanical environment by PDAC cells. Briefly, cells were dissociated from tissue culture plastic and a dilute suspension of cells in microgel was added to either soft (blue) or stiff (pink) 3D microgel culture. After 6 h, cells were isolated via centrifugation and added to 3D microgel culture as shown for an additional 18 h. Cells were extracted after 0, 6, and 24 h in 3D microgel culture, and libraries were prepared for RNA sequencing for each condition.

**Figure 5:**
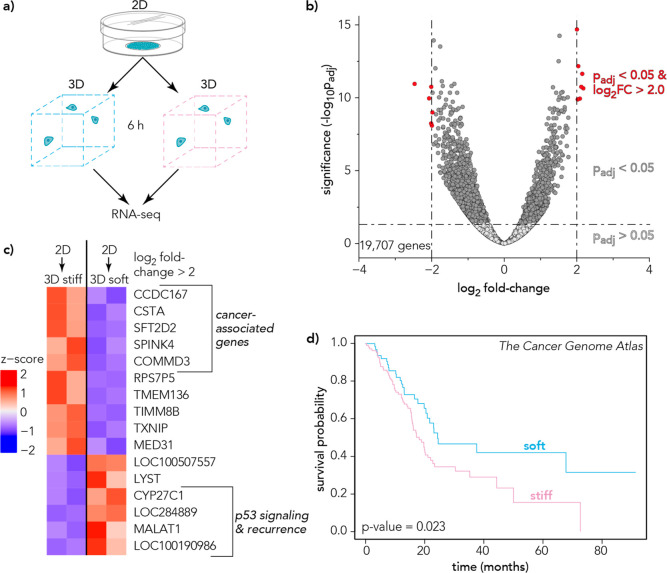
(a) Schematic showing cells removed from tissue culture plastic and cultured for 6 h in a 3D microgel microenvironment under either soft or stiff conditions. (b) Volcano plot of bulk RNA-sequencing results showing differentially expressed genes. Light gray points represent genes with nonsignificant *p*-values. Dark gray points represent genes with significant *p*-values. Points in red represent genes with significant *p*-values and absolute value log_2_ fold-change >2.0. The *p*-values were adjusted using the Benjamini and Hochberg method.^49^ (c) Differentially expressed genes associated with pancreatic cancer comparing cells cultured in soft or stiff 3D microgel culture for 6 h. Different colors represent relative changes in the expression of each gene. Red bins represent upregulated genes, and blue bins represent downregulated genes. (d) Survival probability data are shown for *n* = 177 pancreatic cancer patient samples from The Cancer Genome Atlas (TCGA) using the top differentially expressed genes (log_2_ fold-change >2.0) in PDAC cells after 6 h in stiff microgel culture. Patients with mRNA expression similar to the soft-cultured groups had a mean survival of 24 months, and patients with mRNA expression similar to the stiff-cultured groups had a mean survival of 18 months. Logrank (Mantel–Cox) *p*-value = 0.023.

Pancreatic cancer is a highly metastatic disease.^5^ In order for metastasis to form, a cell or group of cells must migrate from the primary tumor site. We hypothesized that PDAC cells would quickly respond to changes in physiologically relevant stiffness in a 3D culture. To test this hypothesis, PDAC cells were physically moved from 2D tissue culture plastic to either a soft or stiff 3D microgel culture (Figures 4 and 5a, and S3) for 6 h. Cells were isolated for RNA sequencing after 6 h, and differential expression analysis revealed genes with significant changes in expression between soft-cultured cells and stiff-cultured cells (Figure 5b). To address whether the 2D culture plate significantly influences gene expression, we compared cells cultured on conventional tissue culture plates (polystyrene) to cells cultured within the 3D microgel platform, which showed anticorrelated gene expression (Figure S8). We examined top differentially expressed genes in soft vs stiff microgel culture (absolute value log_2_ fold-change >2.0, *p*-value <0.05) for their relation to previously identified genes associated with aggressive cancer. We observed upregulation of *CCDC167*, *CSTA*, *SFT2D2*, *SPINK4*, and *COMMD3* in stiff 3D culture after 6 h compared to soft 3D culture, and previous studies have identified the involvement of these genes with driving cancerous phenotypes (Figure 5c).^55−59^

We examined the effects of these genes in patient data from The Cancer Genome Atlas (TCGA).^60^ Separating the pancreatic cancer patient data from TCGA (*n* = 177) into two groups based on mRNA expression of top differentially expressed genes (Figure 5c) showed changes in overall survival (Figure 5d). Patients with higher mRNA expression of the genes upregulated in stiff 3D culture had a median survival of 18 months compared to 24 months for patients with lower expression of these genes, similar to the soft-cultured PDAC cells (low expression). Additionally, we identified *CYP27C1*, LOC284889, *MALAT1*, and *LOC100190986* as downregulated genes in stiff 3D culture compared to soft 3D culture after 6 h, which are correlated with recurrence or involved in the *p53* signaling pathway.^61−64^

**Figure 6:**
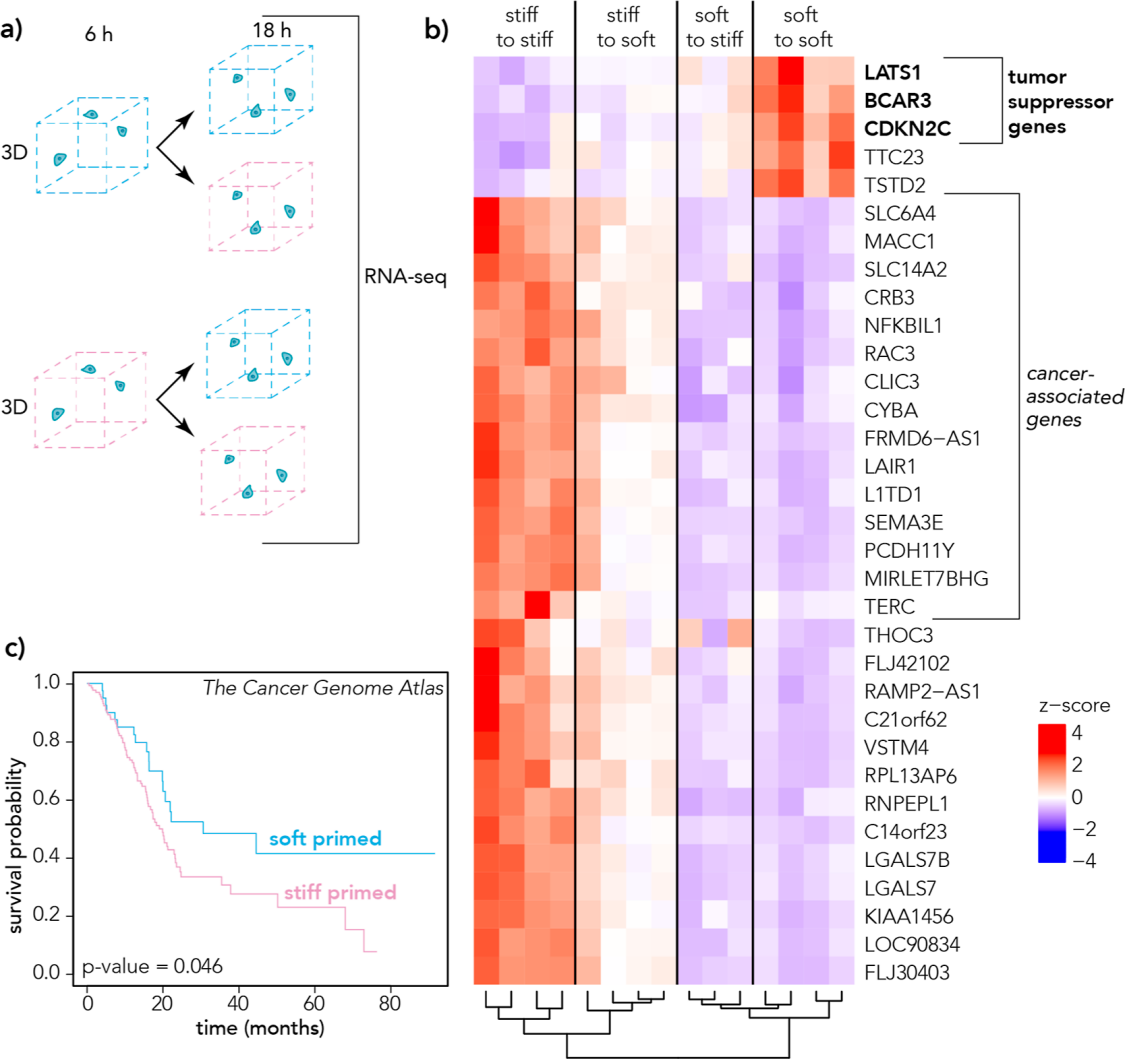
(a) Schematic showing cells were moved from one 3D microgel (priming) environment after 6 h to another 3D microgel (final) environment for 18 h. (b) Differentially expressed genes associated with pancreatic cancer comparing cells cultured for 24 h in a 3D microgel culture system. Cells were switched after 6 h from either soft or stiff microgel to soft or stiff microgel (soft-to-soft, soft-to-stiff, stiff-to-soft, stiff-to-stiff). Hierarchical clustering shows the quality of replicates. (c) Survival probability data are shown for *n* = 177 patient samples from The Cancer Genome Atlas (TCGA) using the top upregulated genes (*n* = 28) in stiff-primed PDAC cells after 24 h in 3D microgel culture. Patients with mRNA expression similar to the soft-primed group had a mean survival of 30 months, and patients with mRNA expression similar to the stiff-primed groups had a mean survival of 19 months. Logrank (Mantel–Cox) *p*-value = 0.046.

We investigated the persistence of mechanical priming in PDAC cells by quantifying their transcriptomics in a 3D microgel culture. PDAC cells were primed for 6 h in either soft or stiff 3D microgels and then each group was switched to either soft or stiff 3D microgel for 18 h (Figure 4). Cells were isolated, and libraries were prepared for RNA sequencing. Our results suggest that priming conditions direct the persistence of gene expression. Figure 6b shows similar gene expression profiles for (i) cells cultured in stiff 3D conditions (“stiff to stiff”) and cells moved from stiff to soft 3D culture (“stiff to soft”) and (ii) cells cultured in soft 3D conditions (“soft to soft”) and cells moved from soft to stiff 3D culture (“soft to stiff”). Remarkably, the top three downregulated genes in conditions where cells experienced a stiff 3D microenvironment for any length of time were tumor suppressor genes *LATS1*, *BCAR3*, and *CDKN2C*.^65−67^ Of the top upregulated genes in stiff-primed conditions (log_2_ fold-change >2, *p*-value < 0.05), several of these genes have been identified as prognostic markers in cancer, including *SLC6A4*, *MACC1*, *SLC14A2*, *CRB3*, *NFKBIL1*, *RAC3*, *CLIC3*, *CYBA*, *FRMD6-AS1*, *LAIR1*, *L1TD1*, *SEMA3E*, *PCDH11Y*, *MIRLET7BHG*, and *TERC* (Figure 6b).^68−81^ We performed GO Term analysis comparing cells primed in soft culture vs cells primed in stiff culture and note changes in terms including transcriptional misregulation in cancer, chromatin organization, nucleus organization, and metabolism of RNA, which may suggest epigenetic mechanisms act as a component responsible for some of these transcriptional changes (Figure S9).^54^ For the genes that may be correlated with mechanical memory, we examined TCGA for pancreatic cancer patient prognosis data.^60^ Separating patients (*n* = 177) into two groups based on mRNA expression of the differentially expressed genes (Figure 6b), patients with a high expression of the genes identified as upregulated in stiff-primed cells had significantly decreased survival (Figure 6c). Patients with low expression of these genes (soft-primed) had a median survival of 30 months, compared to 19 months for patients with expression of these genes similar to stiff-primed cells (high expression).

### 3.4 Mechanical Priming Directs Persistent Protein Expression

**Figure 7:**
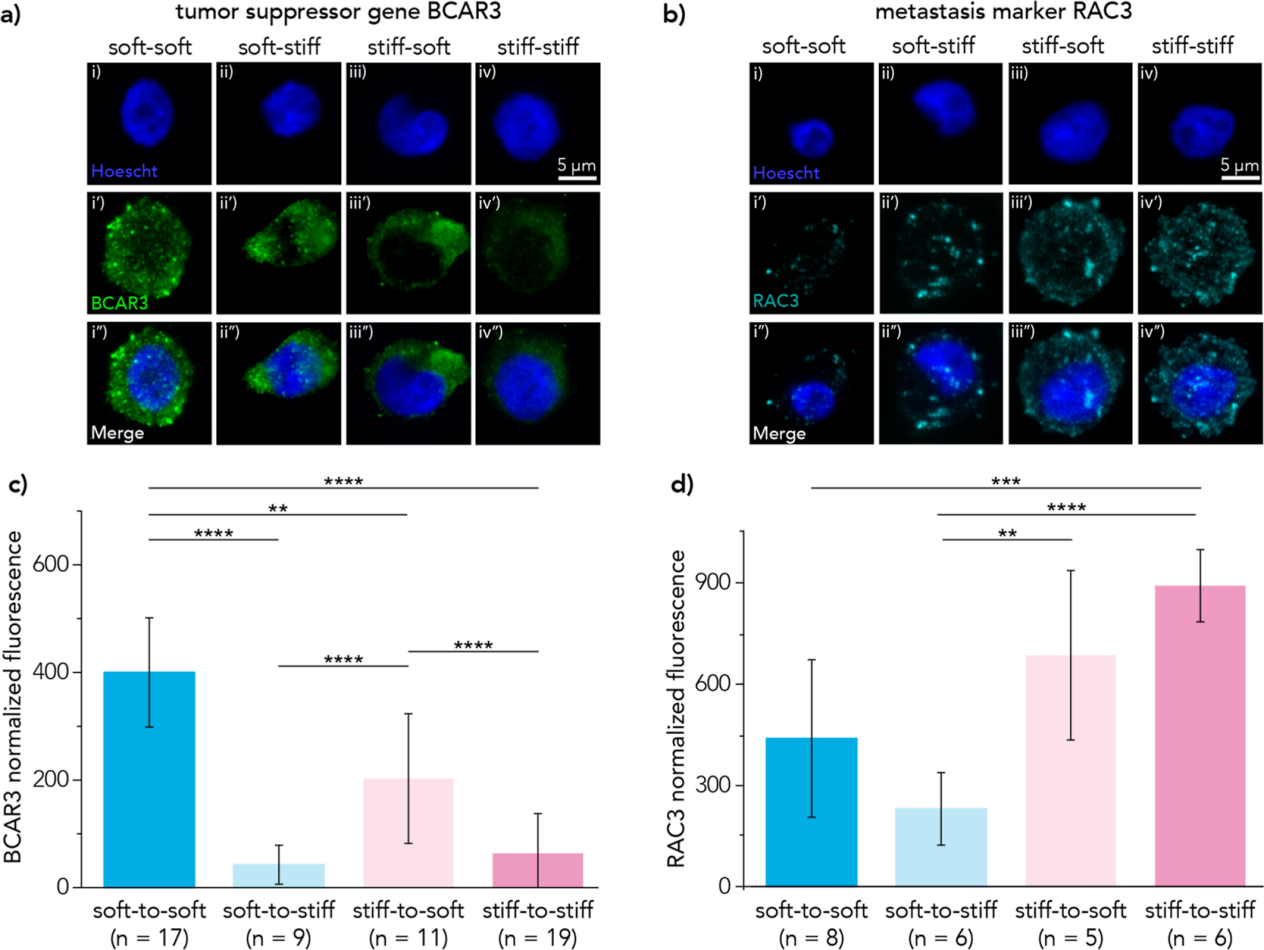
(a) Representative sum-intensity z-projections of immunofluorescence imaging provide additional evidence of persistent mechanical conditioning for the tumor suppressor gene *BCAR3*. (b) Representative sum-intensity z-projections of the metastasis marker *RAC3* are shown to further support protein conversion from RNA-based analyses. (c) Quantification of sum-intensity z-projection fluorescence is shown for the tumor suppressor gene *BCAR3*. Raw fluorescence intensity was normalized to cell area. Significance was calculated using the mean, standard deviation, and number of replicates with a Student’s *t*-test. (d) Quantification of sum-intensity z-projection fluorescence is shown for the metastasis-related gene, *RAC3*. Raw fluorescence intensity was normalized to the cell area, and means and standard deviations are shown. Significance was calculated using a Student’s *t*-test. (*p*-values are denoted as * < 0.05, ** < 0.01, *** < 0.001, **** < 0.0001).

To support the persistence of cellular responses to mechanical priming at the protein level, we performed immunostaining on two of the most differentially expressed genes correlated with mechanosensation after switching microenvironments (soft and stiff). The tumor suppressor gene, *BCAR3*, was found to have decreased expression at the RNA level for cells cultured within the stiff environment compared to cells cultured within the soft environment for 24 h (Figure 6b). Raw intensity values were measured for a sum-intensity z-projection and normalized to the cell area. BCAR3 fluorescence intensity was significantly lower in cells cultured in the stiff environment for 6, 18, or 24 h compared to those cultured entirely in soft 3D culture (Figure 7a,c). We also examined *RAC3*, a gene that was upregulated in stiff-primed conditions (Figure 6b). Cells primed in stiff environments maintained a higher RAC3 expression compared to cells primed in soft conditions (Figure 7b). Sum-intensity projections on the *z*-axis were used to measure raw fluorescence intensity values, which were normalized to cell area measurements. Cells primed in stiff conditions expressed statistically (*p* < 0.01) higher levels of RAC3 compared to those primed in soft conditions (Figure 7d). Additional evidence of mechanosensation was observed as differences in radial acetylated-tubulin expression,^82−87^ with soft-cultured cells exhibiting membrane-localized tubulin and stiff-cultured cells exhibiting tubulin expression throughout the cell body after 24 h (Figures S10 and S11). Microtubule acetylation has been shown to play a role in mechanosensitive adhesion and migration through focal adhesions and YAP translocation and can promote traction forces via actomyosin contractility.^82,84^ Microtubules have also been shown to regulate nuclear invaginations, and these precise shape changes have been shown to direct loss of chromatin accessibility, changes in gene expression, and phenotypic changes.^85−87^ Together, our results support our hypothesis that PDAC cells cultured in 3D microgel environments maintain persistent cellular responses to mechanical conditioning at both the RNA and protein levels.

## 4 Conclusions

We demonstrate that polyacrylamide hydrogels can be designed to emulate the mechanical microenvironments of healthy and cancerous pancreatic tissue, and microgels engineered from these hydrogels exhibit tunable rheological properties. Our 3D microgel platform was used to support short-term (<24 h) culture of human pancreatic ductal adenocarcinoma cells. Cells were primed in either soft or stiff 3D culture for 6 h and transferred to soft or stiff 3D culture for another 18 h prior to RNA-seq analysis to investigate the extent to which cellular responses in cancer are sensitive to subtle changes in the mechanical properties of the microenvironment. Our results indicate that PDAC cells primed in stiff 3D culture in vitro overexpress genes associated with cancer, *p53* signaling, and cancer recurrence. In contrast, the gene expression profiles of PDAC cells primed in soft 3D microgel culture were correlated with lower expression of prognostic markers of cancer and higher expression of genes associated with tumor suppression. Even acute priming events (6 h) in stiff 3D culture were sufficient to direct gene expression for up to 24 h. Our results suggest that PDAC cells maintain persistent cellular responses to mechanical priming, even when cultured in two different 3D culture conditions that are both softer than a single cell. Key genes identified by RNA-seq associated with cancer prognostic markers were compared against The Cancer Genome Atlas and revealed significant differences in survival probability between soft-primed and stiff-primed 3D culture (*p*-value <0.05). Two key genes identified using RNA sequencing as being involved with persistent cellular priming were validated with immunofluorescence staining, supporting this maintenance at the protein level. Cancer-associated genes identified herein may be dependent upon the mechanical and rheological properties of the tumor microenvironment; our future work will investigate mechanotransduction mechanisms responsible for differences in growth rates between slow-growing small tumors surrounded by soft healthy tissue and fast-growing larger tumors confined by stiffer diseased tissue. This microgel system can be leveraged to further understand mechanical memory in PDAC by modifying cytoskeletal functions, probing chromatin accessibility and epigenetic changes, and exploring transcription factor activity. Mechanical memory in PDAC could be exploited by future therapeutic strategies designed to soften the tumor microenvironment and improve prognosis.

## Data Availability

The data that support the findings of this study are openly available in Dryad at https://doi.org/10.5061/dryad.z34tmpgn8.

## Supporting Information Available

Additional experimental details and materials including images of microgel on various length scales (Figure S1), size distribution of microgel particles (Figure S2), color-height maps of microgel and PDAC cells (Figure S3), cell viability (Figure S4), Pearson correlation coefficients of RNA-seq replicates (Figure S5), packing density and channel dimension images (Figures S6 and S7), heatmap comparing 2D plastic culture to 3D culture (Figure S8), GO Term analysis (Figure S9), and radial tubulin expression images and analyses (Figures S10 and S11) (PDF)
