# Supplementary material for: Survival-Associated Cellular Response Maintained in Pancreatic Ductal Adenocarcinoma (PDAC) Switched Between Soft and Stiff 3D Microgel Culture
Source: ACS Biomater Sci Eng. 2024 Mar 11;10(4):2177–87. doi: 10.1021/acsbiomaterials.3c01079 (PMC11005012; doi:10.1021/acsbiomaterials.3c01079)
Supplement: Supplementary file 1 — ab3c01079_si_001.pdf [file ab3c01079_si_001.pdf]

Supplementary Materials:
Survival-Associated Cellular Response Maintained in Pancreatic Ductal Adenocarcinoma (PDAC) Switched Between Soft and Stiff 3D Microgel Culture

[1]Dixon J. Atkins, [1]Jonah M. Rosas, [2]Lisa K. Månsson, [3]Nima Shahverdi, [4,5*]Siddharth S. Dey, and [2*]Angela A. Pitenis

[1]Department of Biomolecular Science and Engineering
[2]Materials Department
[3]Molecular, Cellular, and Developmental Biology Department
[4]Department of Chemical Engineering
[5]Department of Biological Engineering
University of California, Santa Barbara
Santa Barbara, CA, 93106, USA

*Corresponding authors:
Siddharth S. Dey (sdey@ucsb.edu) and Angela A. Pitenis (apitenis@ucsb.edu)

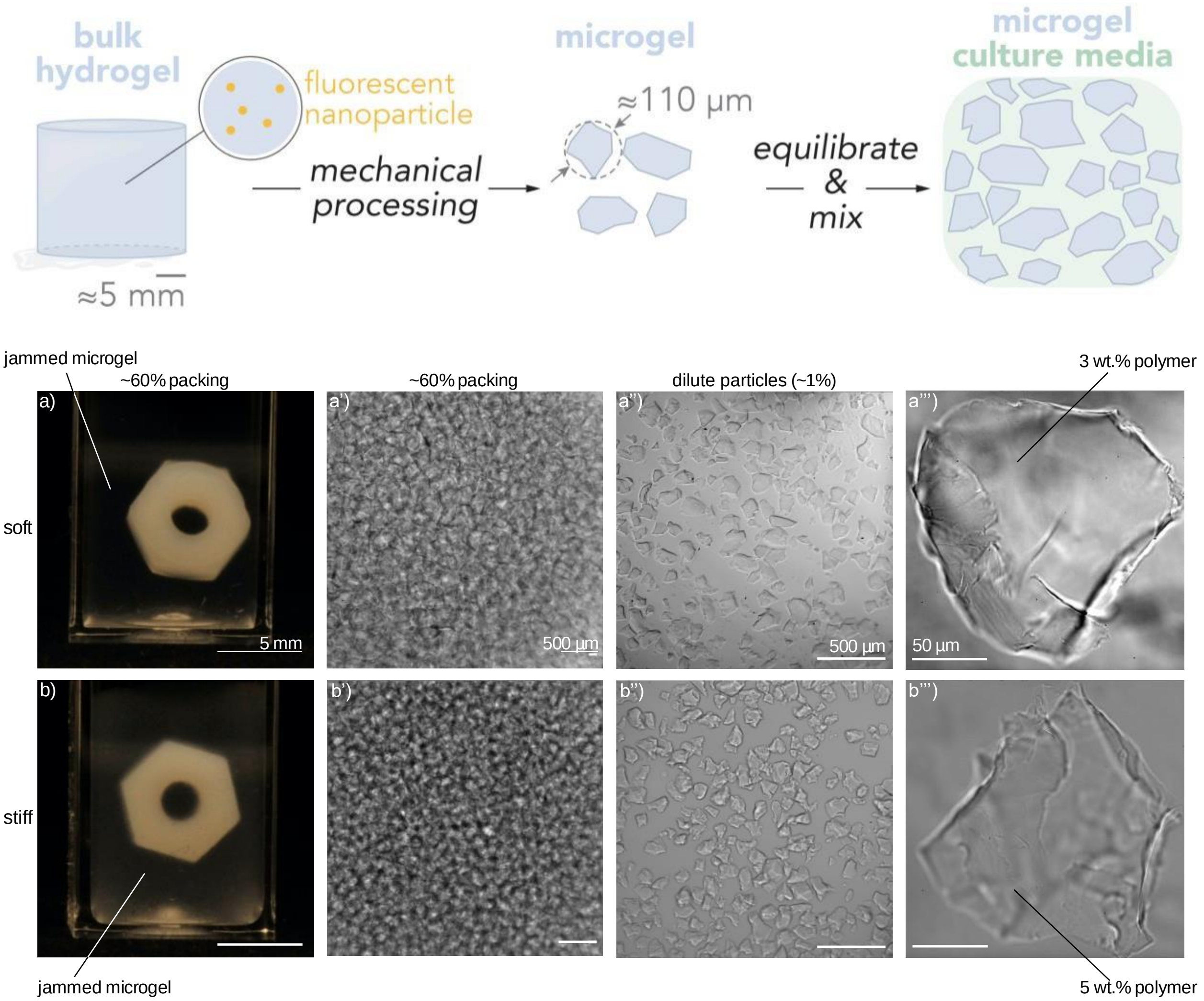

Figure S1: Schematic of microgel processing procedure (top). Macroscale view of (a) soft and (b) stiff jammed microgel in a glass cuvette supporting a nylon nut in 3D. Scale bars = 5 mm. (a′,b′) Single z-plane of jammed microgel (about 60% packing fraction) in brightfield. Scale bars = 500 µm. (a′′,b′′) Single z-plane of low concentration of soft and stiff microgel particles (about 1% packing fraction) in brightfield to show size distribution. Scale bars = 500 µm. High magnification brightfield images of a (a′′′) single soft microgel particle composed of crosslinked 3 wt.% polyacrylamide hydrogel and (b′′′) single stiff microgel particle composed of crosslinked 5 wt.% polyacrylamide hydrogel. Scale bars = 50 µm.

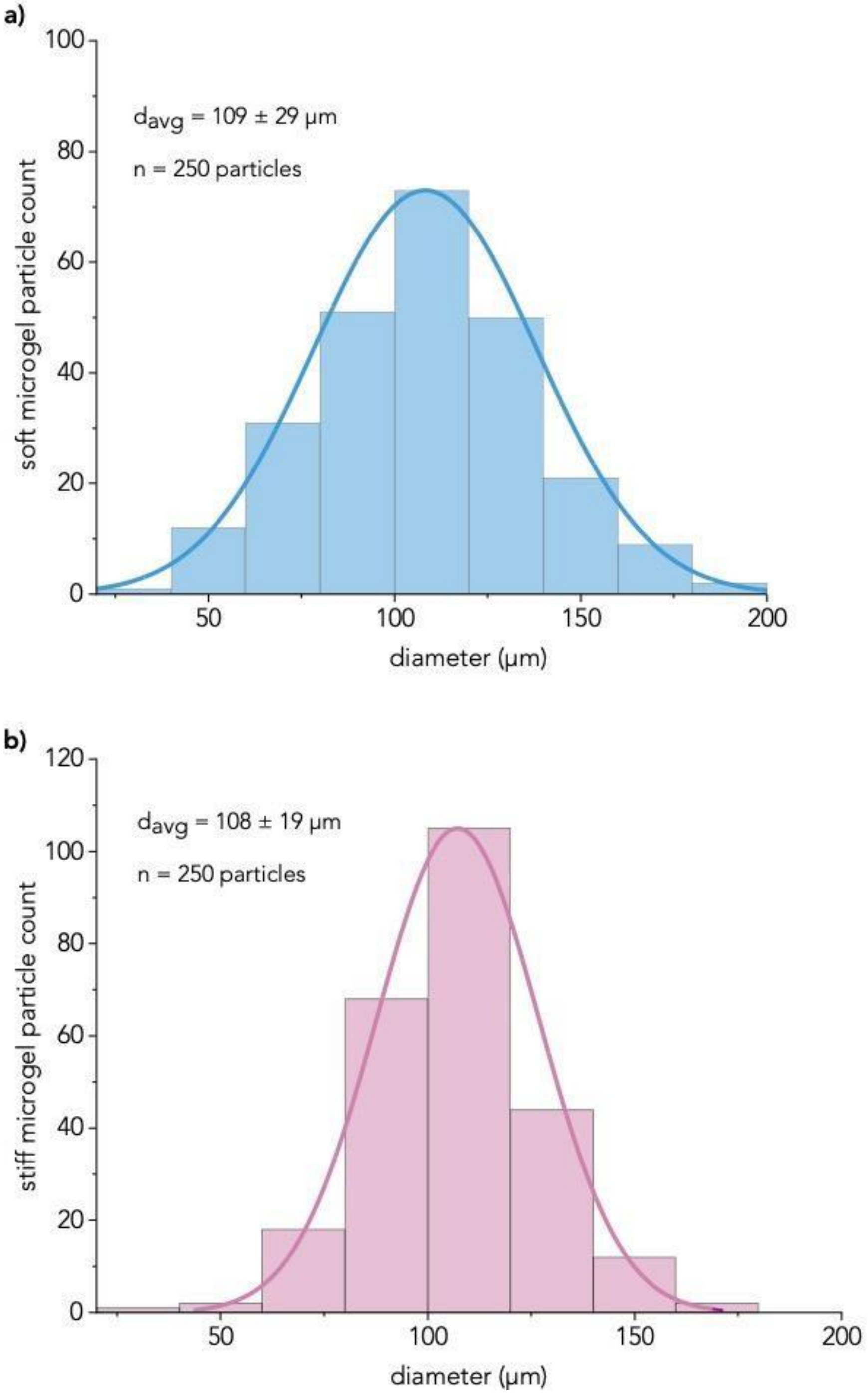

Figure S2: Distribution histograms of representative samples of (a) soft microgel and (b) stiff microgel with an average size distribution of 109 µm ± 29 µm and 108 µm ± 19 µm, respectively. Each distribution was calculated as an average of 250 particles as shown in Figure S1 a'' and b''.

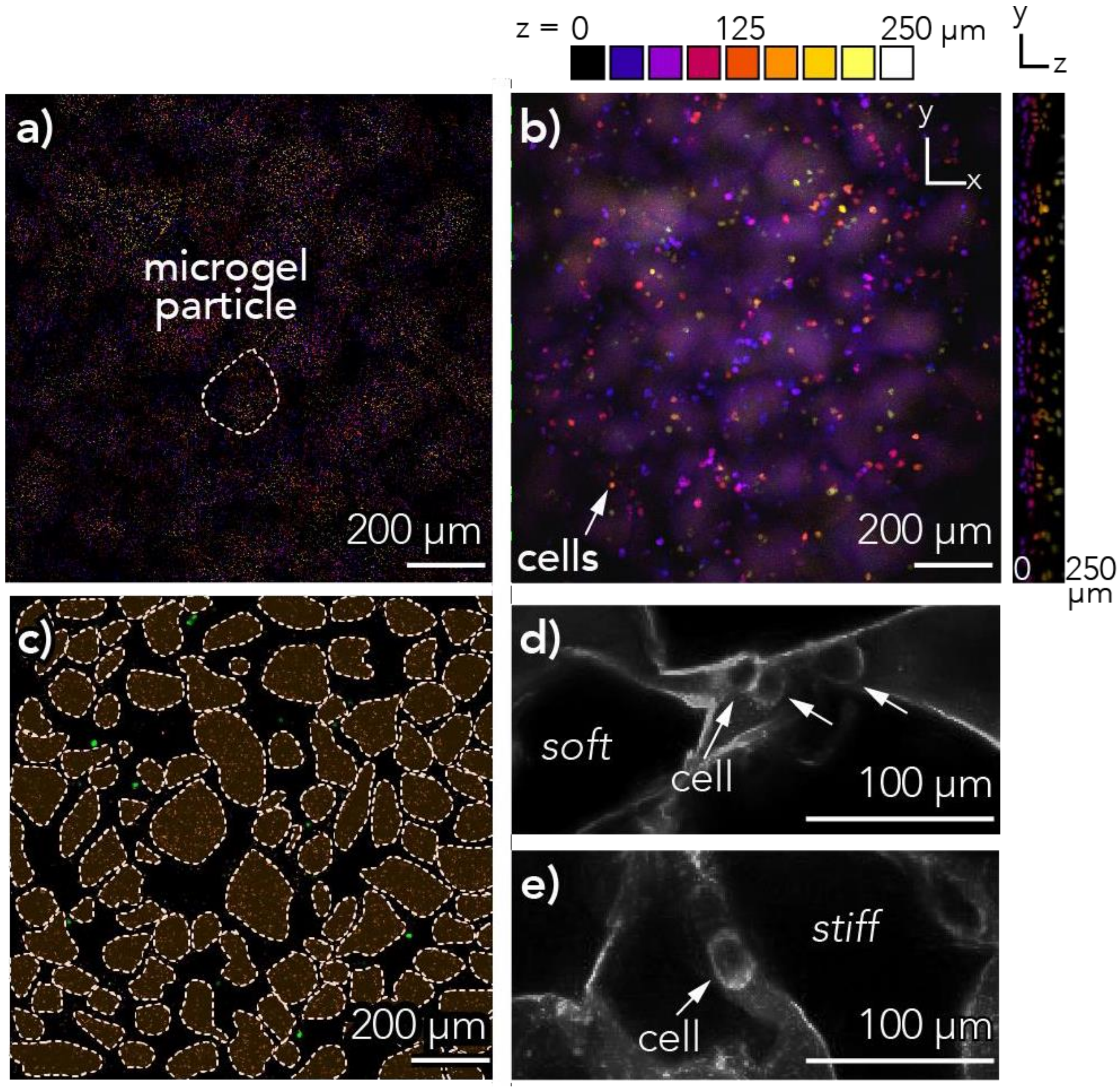

Figure S3: (a) Confocal image processed into a color-height map of fluorescent jammed microgel particles (orange). One microgel particle is outlined to show detail. (b) Confocal image of the same color-height map microgel now including cells (stained with CellTracker green) supported within different z-planes in the 3D microgel system. Both microgels and cells are visible in the y-x image, while only cells are visible in the y-z image. (c) One z-slice of the same confocal image from (a) and (b) shows microgel (false colored orange and with dashed outlines) and cells (green) on one plane. Monochromatic fluorescence image shows cells (denoted by arrows) in a channel between (d) soft and (e) stiff microgel particles. The cell between two stiff microgel particles appears subjected to some compressive stresses due to confinement.

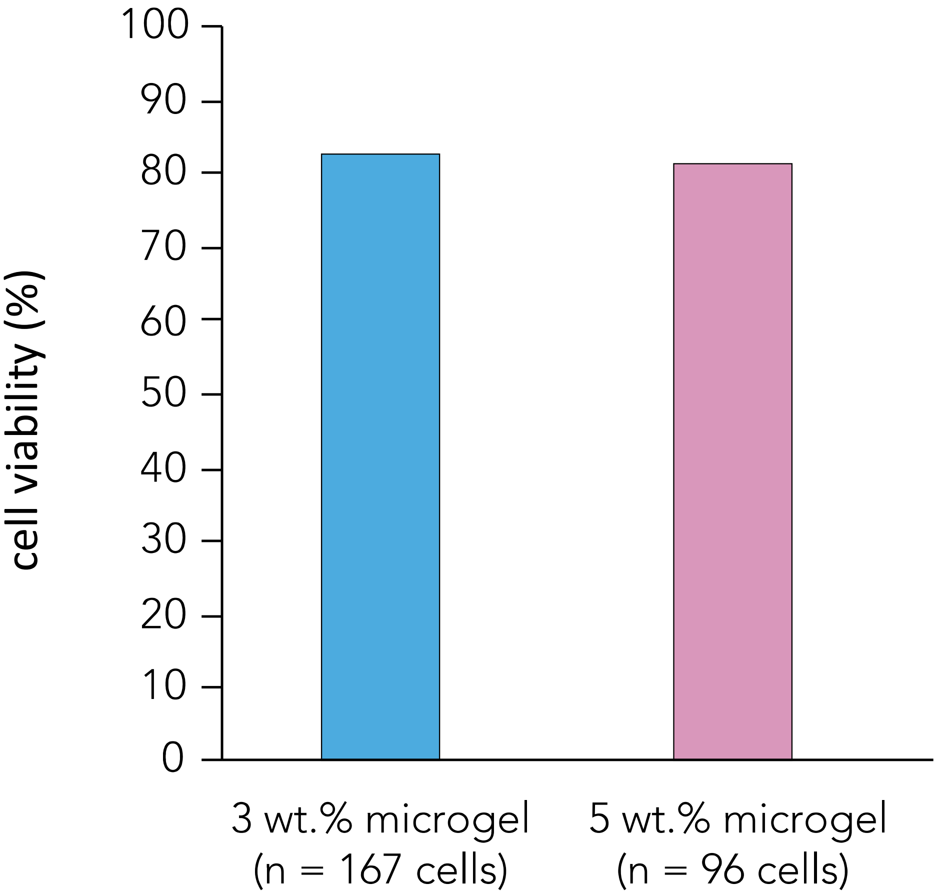

Figure S4: Cell viability was determined using propidium iodide stain after 24 h in 3D microgel culture to be 83% for cells cultured in soft (3 wt.% polyacrylamide) microgel and 82% for cells cultured in stiff (5 wt. % polyacrylamide) microgel.

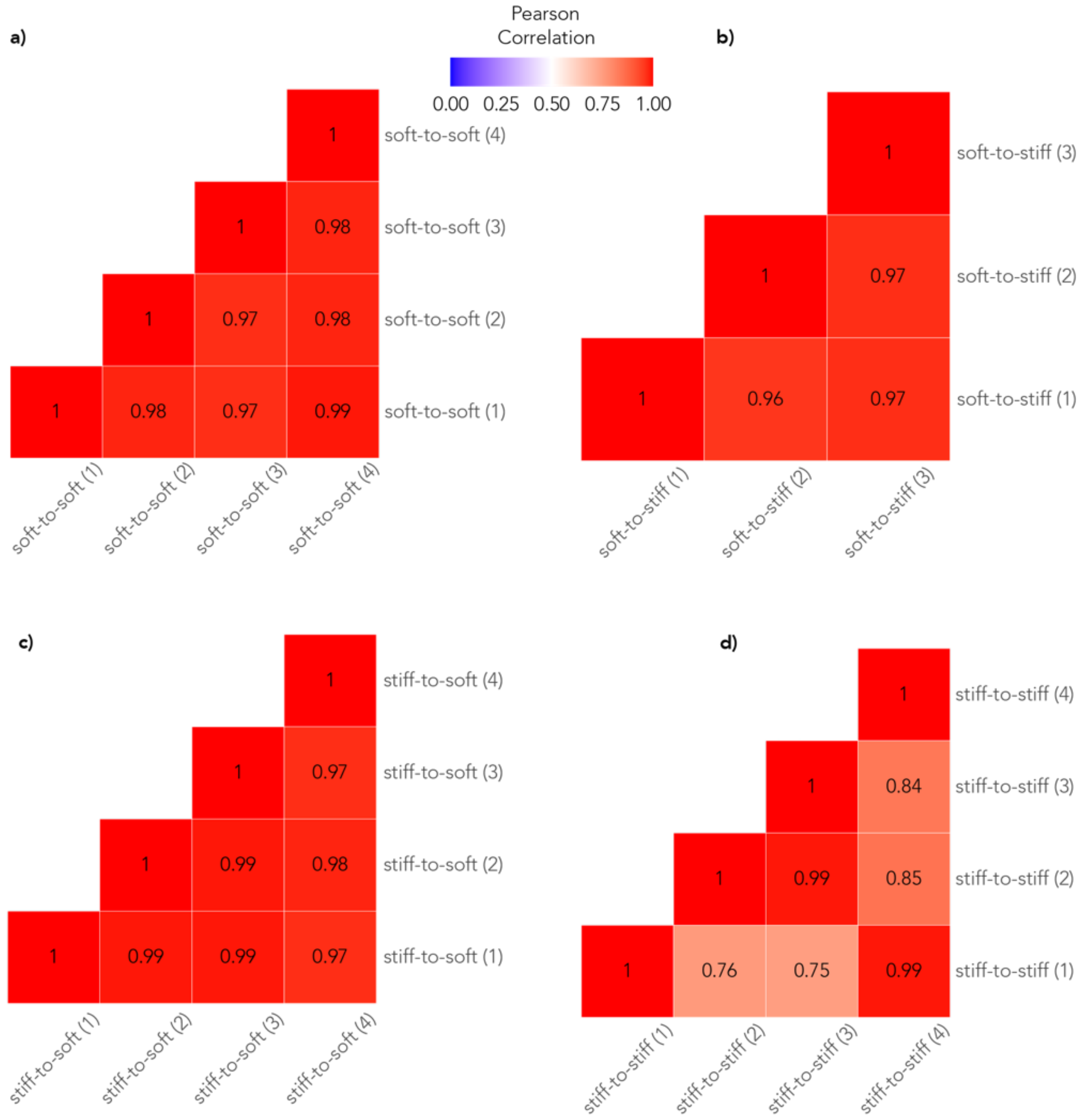

Figure S5: Pearson correlation coefficient heat maps showing linear correlation between replicate samples of (a) soft-to-soft (b) soft-to-stiff (c) stiff-to-soft and (d) stiff- to-stiff conditions.

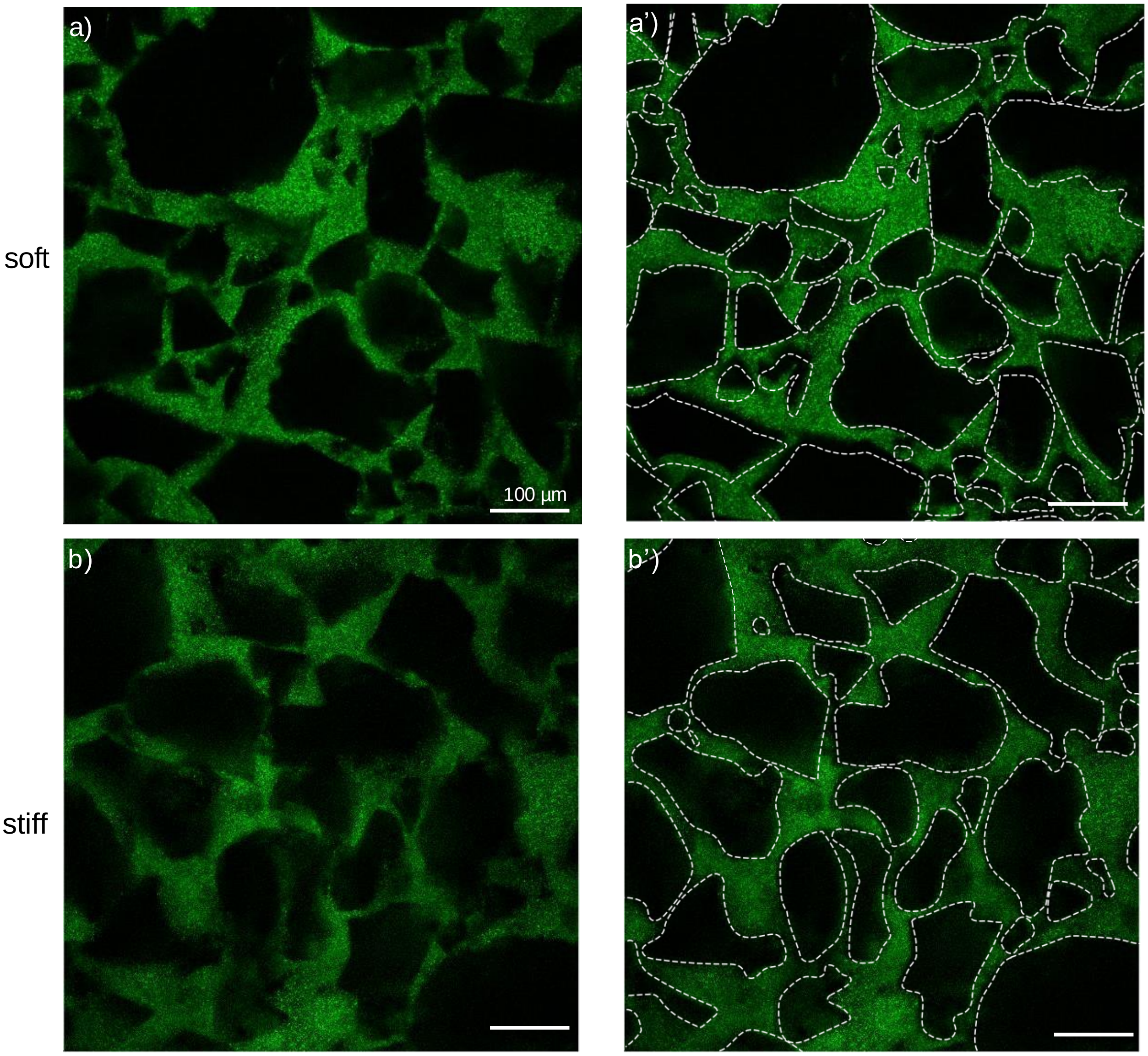

Figure S6: Single z-slice of confocal images of interstitial space (labeled with 100 nm green fluorescent polystyrene beads) between jammed (a, a′) soft and (b, b′) stiff microgel particles to determine microgel packing fraction. (a′, b′) Microgel particles (black, unlabeled) were outlined and their fractional area was used to calculate packing fraction, which was (a′) 61 ± 3% for the soft microgel system (n = 3 slices) and (b′) 57 ± 3% for the stiff microgel system (n = 3 slices). Scale bars = 100 µm.

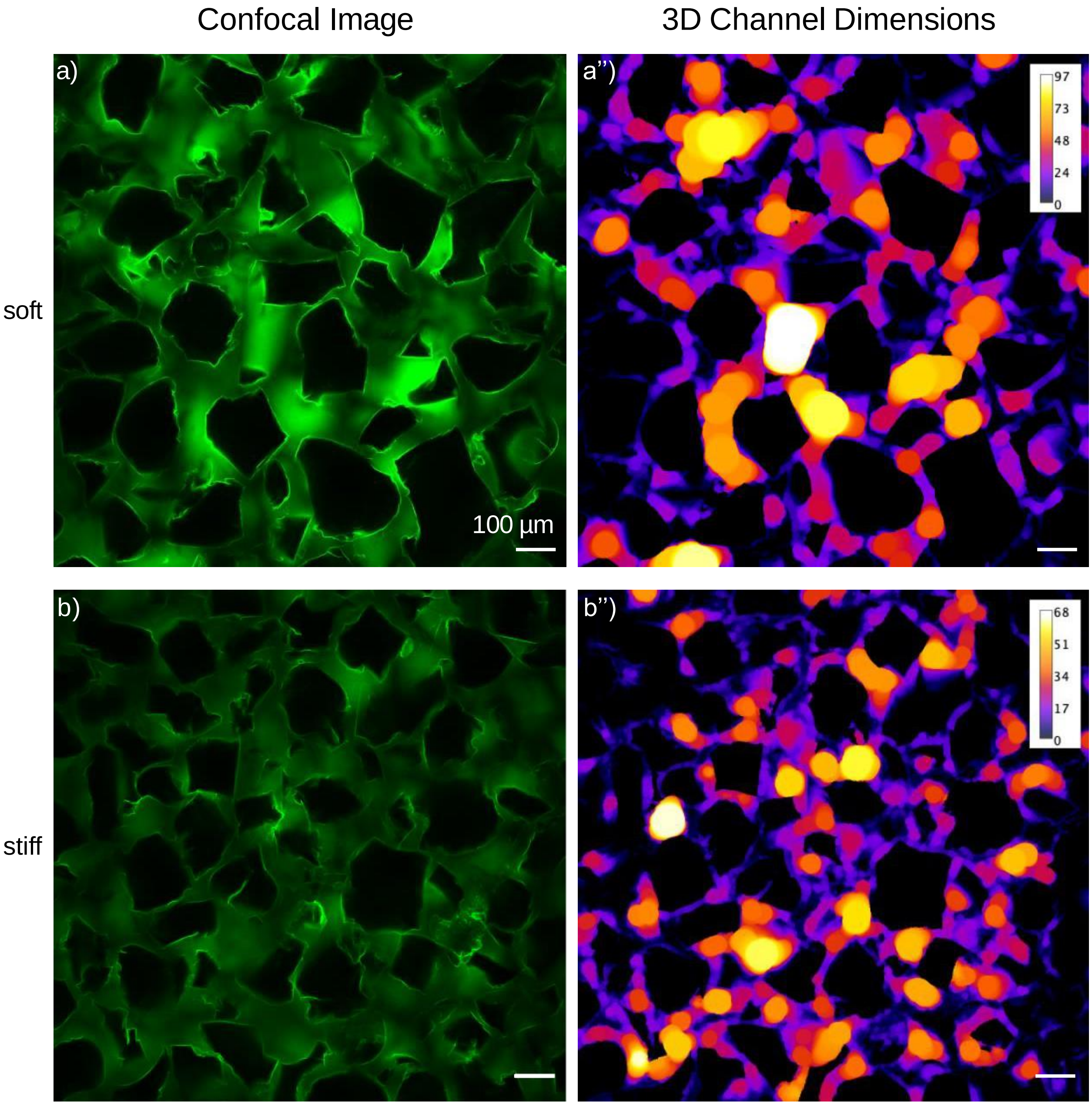

Figure S7: Interstitial spaces between jammed microgel particles were labeled with 100 nm green fluorescent polystyrene beads (Fluoro-Max), and cross sections were analyzed to determine maximum channel diameters. Local thickness was calculated using an overlapping ball algorithm built into FIJI. Raw confocal images of (a) soft and (b) stiff microgels. Local thickness plots for one z-slice give channel widths between (a'') soft and (b'') stiff microgel particles. Channel dimensions of jammed microgels were calculated as (a) 11.7 ± 21 µm for the soft microgel system and (b) 8.5 ± 14 µm for the stiff microgel system. (n = 40 z-slices, 80 µm stack). Scale bars = 100 µm.

a)

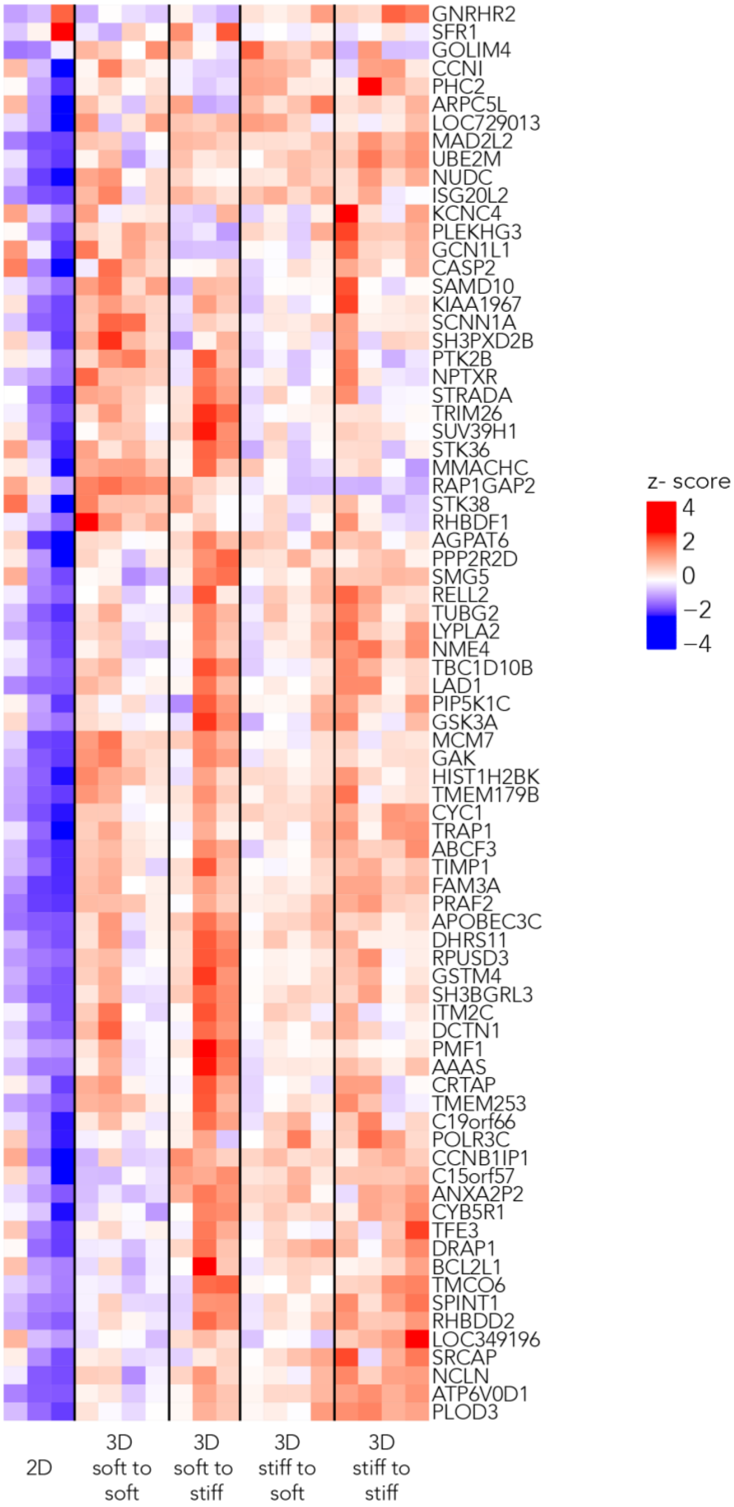
GNRHR2
SFR1
GOLIM4
CCNI
PHC2
ARPC5L
LOC729013
MAD2L2
UBE2M
NUDC
ISG20L2
KCNC4
PLEKHG3
GCN1L1
CASP2
SAMD10
KIAA1967
SCNN1A
SH3PXD2B
PTK2B
NPTXR
STRADA
TRIM26
SUV39H1
STK36
MMACHC
RAP1GAP2
STK38
RHBDF1
AGPAT6
PPP2R2D
SMG5
RELL2
TUBG2
LYPLA2
NME4
TBC1D10B
LAD1
PIP5K1C
GSK3A
MCM7
GAK
HIST1H2BK
TMEM179B
CYC1
TRAP1
ABCF3
TIMP1
FAM3A
PRAF2
APOBEC3C
DHRS11
RPUSD3
GSTM4
SH3BGRL3
ITM2C
DCTN1
PMF1
AAAS
CRTAP
TMEM253
C19orf66
POLR3C
CCNB1IP1
C15orf57
ANXA2P2
CYB5R1
TFE3
DRAP1
BCL2L1
TMCO6
SPINT1
RHBDD2
LOC349196
SRCAP
NCLN
ATP6V0D1
PLOD3
2D
3D soft to soft
3D soft to stiff
3D stiff to soft
3D stiff to stiff
z- score
4
2
0
−2
−4

b)

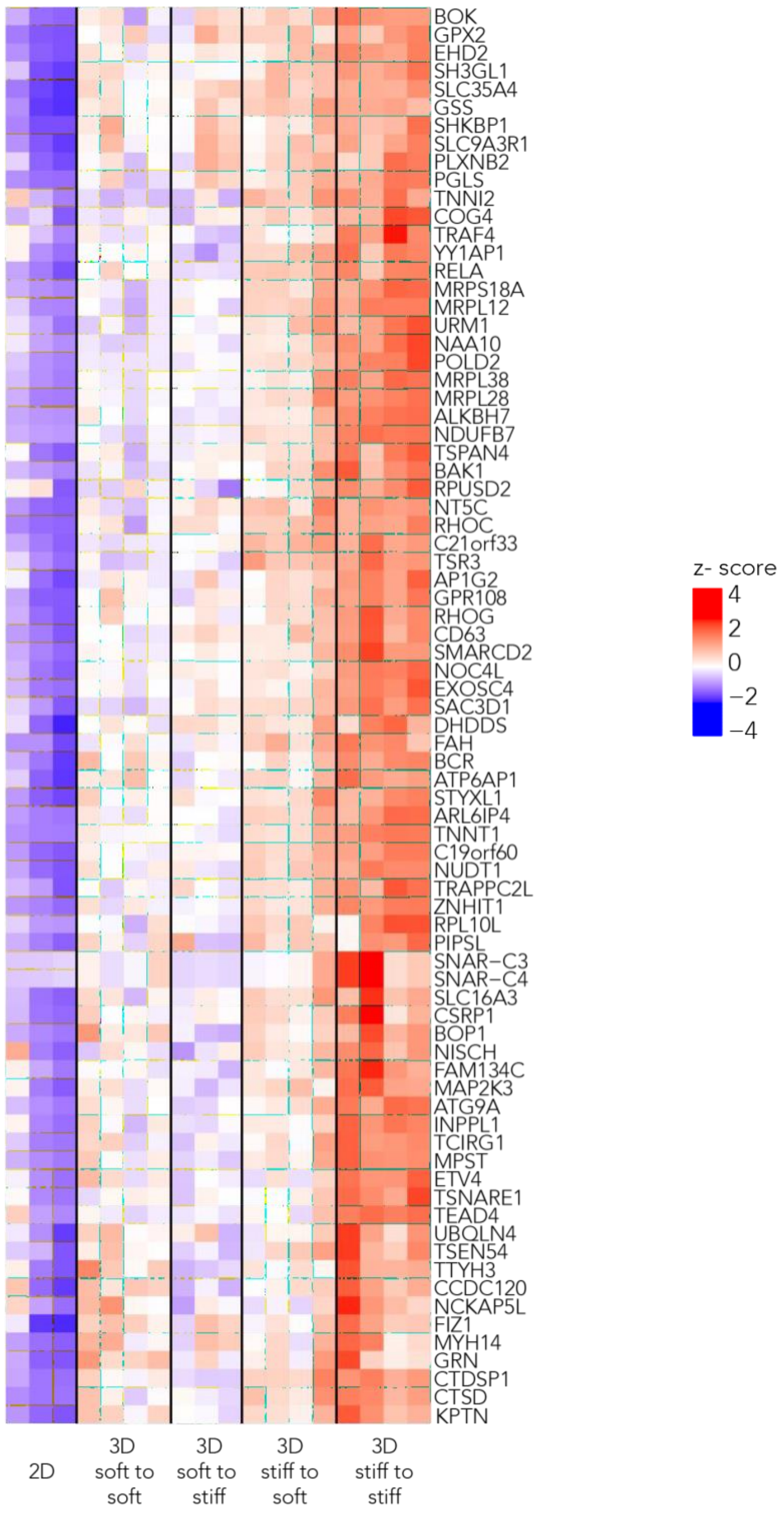
BOK
GPX2
EHD2
SH3GL1
SLC35A4
GSS
SHKBP1
SLC9A3R1
PLXNB2
PGLS
TNNI2
COG4
TRAF4
YY1AP1
RELA
MRPS18A
MRPL12
URM1
NAA10
POLD2
MRPL38
MRPL28
ALKBH7
NDUFB7
TSPAN4
BAK1
RPUSD2
NT5C
RHOC
C21orf33
TSR3
AP1G2
GPR108
RHOG
CD63
SMARCD2
NOC4L
EXOSC4
SAC3D1
DHDDS
FAH
BCR
ATP6AP1
STYXL1
ARL6IP4
TNNT1
C19orf60
NUDT1
TRAPPC2L
ZNHIT1
RPL10L
PIPSL
SNAR−C3
SNAR−C4
SLC16A3
CSRP1
BOP1
NISCH
FAM134C
MAP2K3
ATG9A
INPPL1
TCIRG1
MPST
ETV4
TSNARE1
TEAD4
UBQLN4
TSEN54
TTYH3
CCDC120
NCKAP5L
FIZ1
MYH14
GRN
CTDSP1
CTSD
KPTN
2D
3D soft to soft
3D soft to stiff
3D stiff to soft
3D stiff to stiff
z- score
4
2
0
−2
−4

c)

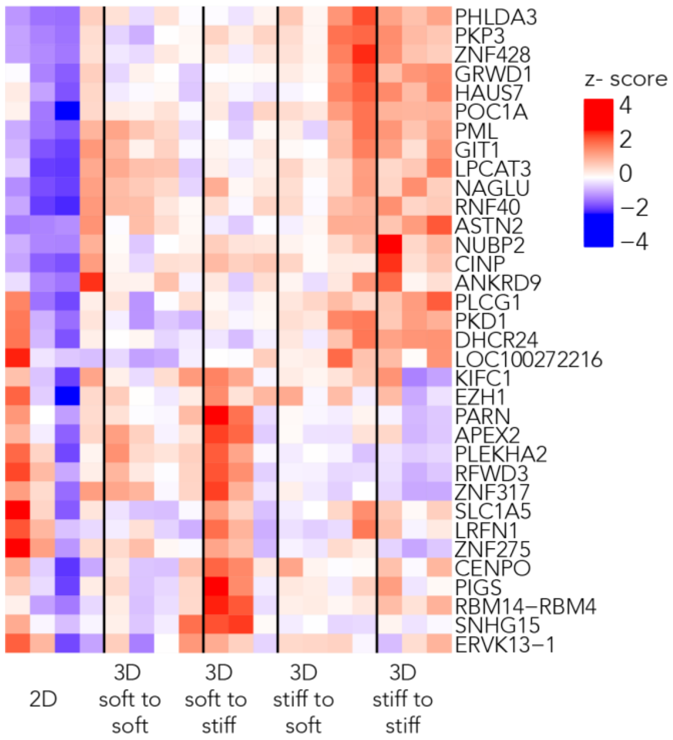

Figure S8: Heat map of top differentially expressed genes (> 1.5$\log_2$fold) shows anti-correlation between PDAC cells cultured on 2D polystyrene tissue culture plates and in 3D microgel (soft and stiff). Map shown above in three parts, in order: (a), (b), and (c).

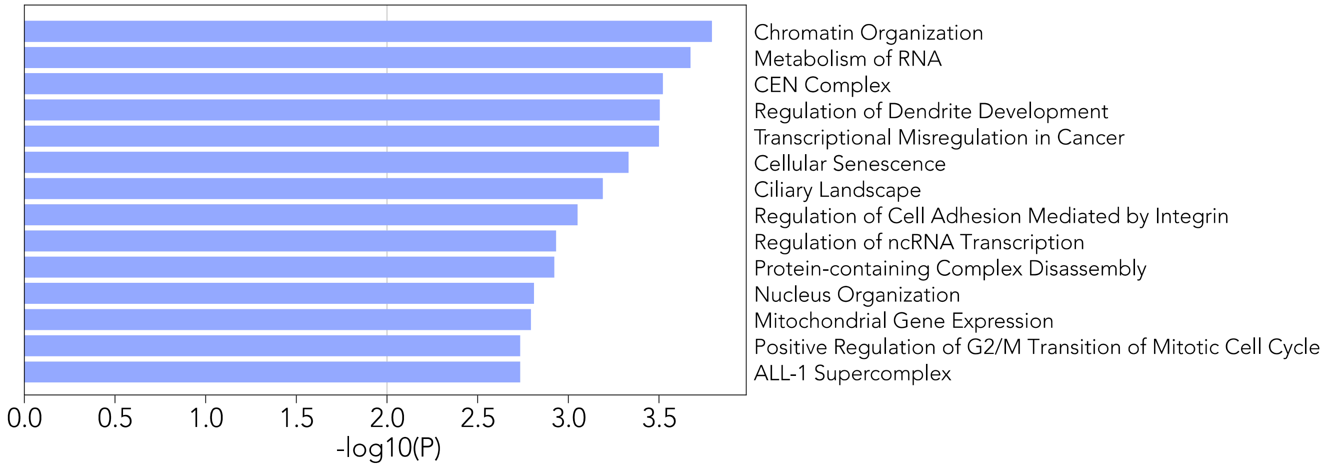

Figure S9: GO Term analysis comparing cells initially primed in soft conditions to those initially primed in stiff conditions after 24 h of 3D microgel cell culture.

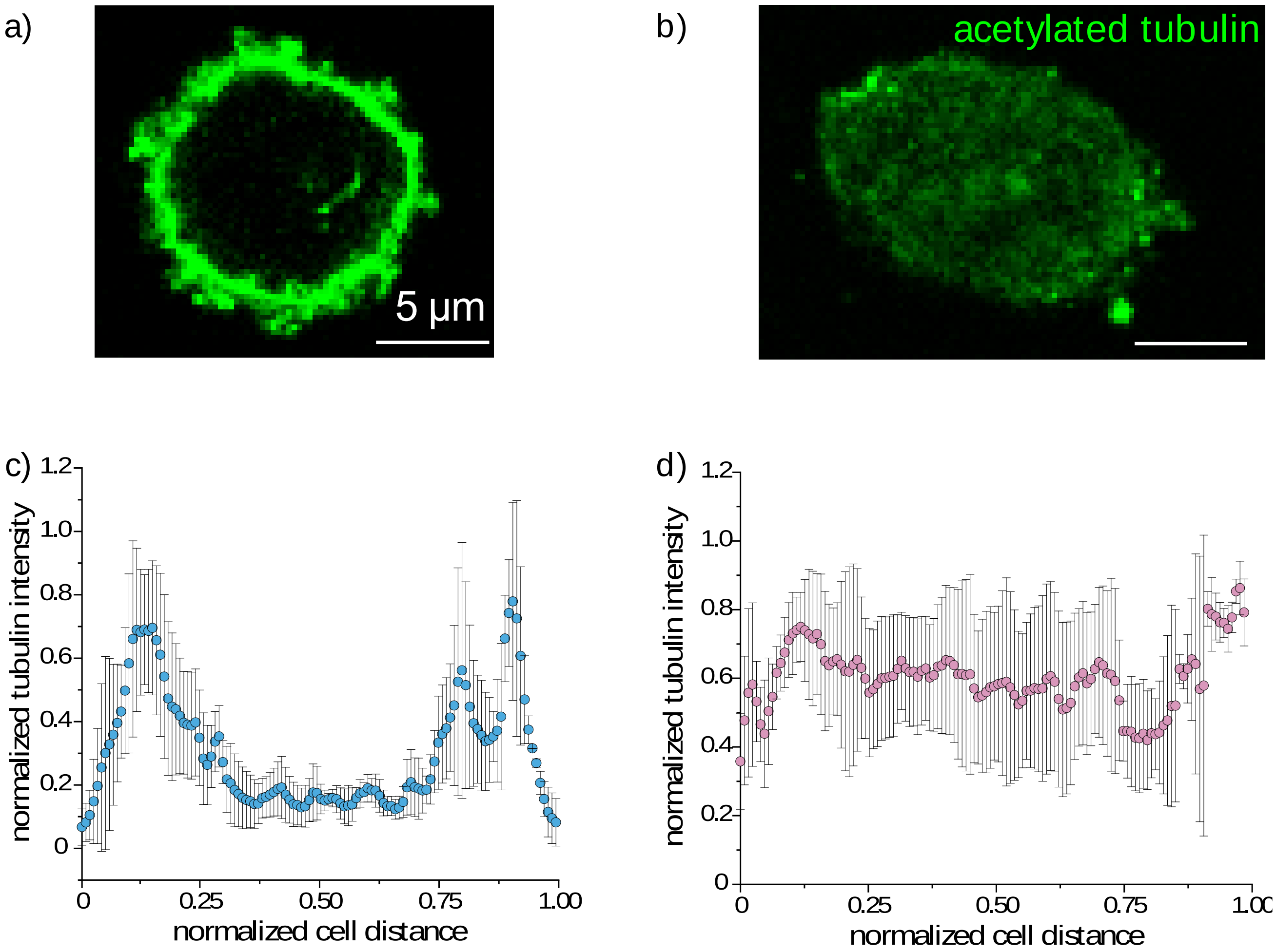

Figure S10: Confocal images of the midplane of single cells. Radial tubulin intensity shown as a mean and standard deviation of n = 4 cells normalized to cell diameter and mean intensity for each cell cultured for 24 h in (a,c) soft microgel or (b,d) stiff microgel. (a,b) Scale bars = 5 µm. Raw images are shown for soft-cultured and stiff-cultured cells in Figure S11 (a) and (b), respectively.

a)

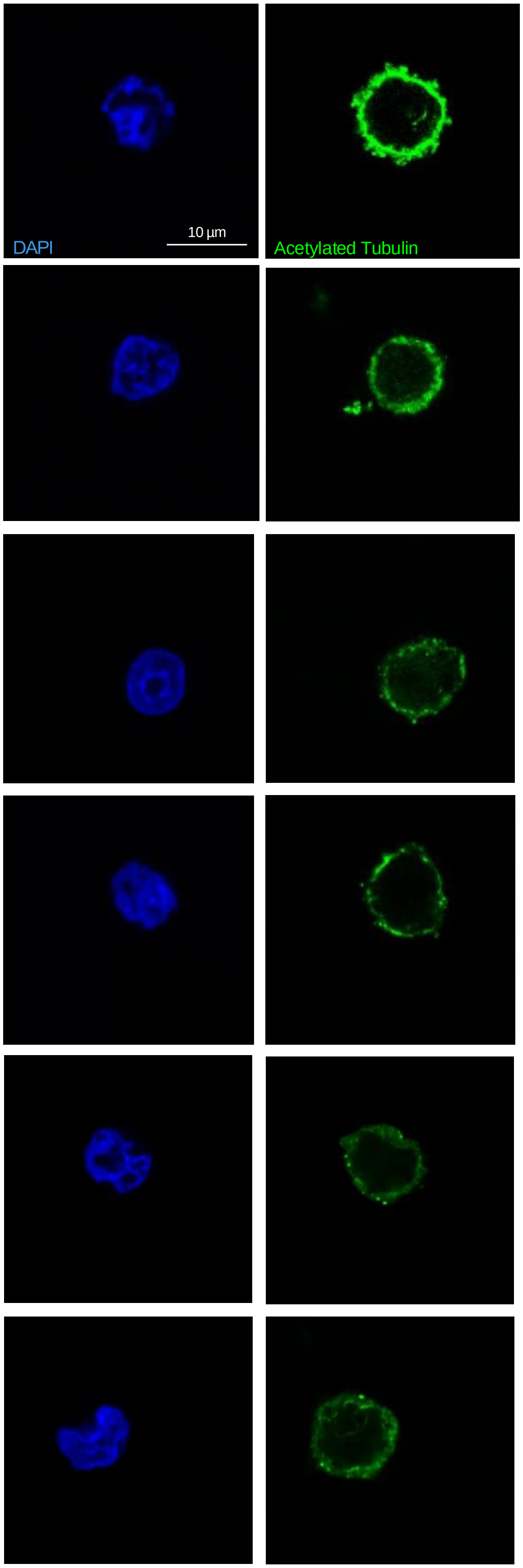

b)

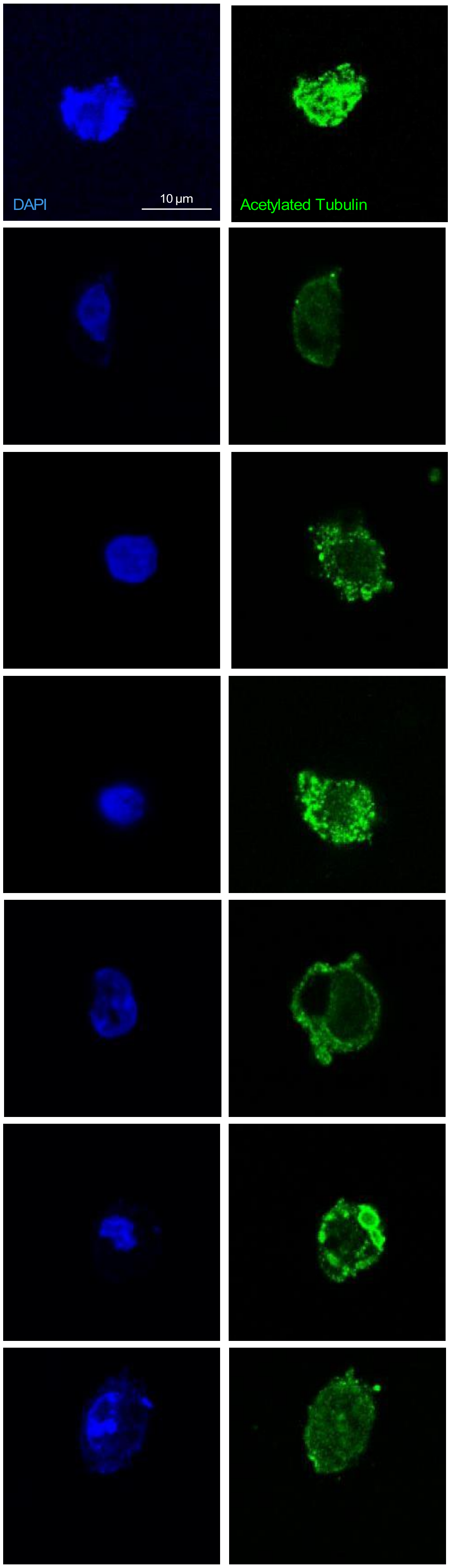

Figure S11: Raw images of data shown in Figure S10 (c) and (d) are shown for soft-cultured and stiff-cultured cells in (a) and (b), respectively.
